# Uniform segmented platelet micelles with compositionally distinct and selectively degradable cores

**DOI:** 10.1038/s41557-023-01177-2

**Published:** 2023-04-20

**Authors:** Zaizai Tong, Yujie Xie, Maria C. Arno, Yifan Zhang, Ian Manners, Rachel K. O’Reilly, Andrew P. Dove

**Affiliations:** 1 College of Materials Science and Engineering, Zhejiang Sci-Tech University, Hangzhou, P. R. China; 2 School of Chemistry, University of Birmingham, Edgbaston, Birmingham, UK; 3 Department of Chemistry, University of Victoria, Victoria, British Columbia Canada; 4 Centre for Advanced Materials and Related Technology (CAMTEC), University of Victoria, Victoria, British Columbia Canada

**Keywords:** Polymers, Molecular self-assembly

## Abstract

The creation of nanoparticles with controlled and uniform dimensions and spatially defined functionality is a key challenge. The recently developed living crystallization-driven self-assembly (CDSA) method has emerged as a promising route to one-dimensional (1D) and 2D core–shell micellar assemblies by seeded growth of polymeric and molecular amphiphiles. However, the general limitation of the epitaxial growth process to a single core-forming chemistry is an important obstacle to the creation of complex nanoparticles with segmented cores of spatially varied composition that can be subsequently exploited in selective transformations or responses to external stimuli. Here we report the successful use of a seeded growth approach that operates for a variety of different crystallizable polylactone homopolymer/block copolymer blend combinations to access 2D platelet micelles with compositionally distinct segmented cores. To illustrate the utility of controlling internal core chemistry, we demonstrate spatially selective hydrolytic degradation of the 2D platelets—a result that may be of interest for the design of complex stimuli-responsive particles for programmed-release and cargo-delivery applications.

**Figure:**
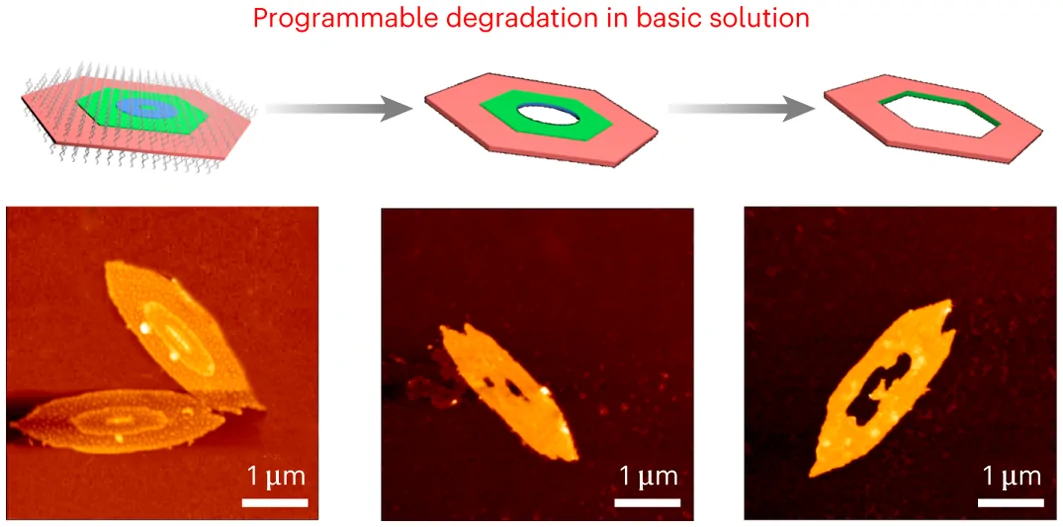

## Abstract (web summary)

The synthesis of two-dimensional nanostructures with controlled dimensions from polymeric precursors remains challenging. Now, two-dimensional nanoparticles with chemically different spatially defined cores have been fabricated through seeded growth and are shown to undergo a programmable degradation process.

## Main

Two-dimensional (2D) nanostructures with precisely controlled compositions and dimensions based on polymeric precursors are of widespread interest, but are challenging to access^1–6^. Living crystallization-driven self-assembly (CDSA) of block copolymers (BCPs) and related polymeric amphiphiles in selective solvents using preformed seeds has been demonstrated to be a powerful, ambient-temperature method for the formation of 2D platelet micelles of controlled size^7,8^. A range of well-defined and complex platelet architectures from BCPs and a variety of particle morphologies have been realized using this approach^9–18^. Precise control of the internal structure of the core–shell particles to allow the creation of cores with spatially distinct chemistries could enable advances in particle behaviour. However, this remains an unresolved challenge due to the general limitation that seeded epitaxial growth leads to a core structure for the resulting particles that is compositionally homogeneous^19–24^. Only limited examples of heteroepitaxial growth have been reported for 1D or 2D assemblies^14,25,26^, and the most extensively studied case involves structurally similar poly(ferrocenyldimethylsilane) (PFS) and poly(ferrocenyldimethylgermane) (PFG) as the crystallizable core-forming blocks. These materials possess near identical physical and chemical properties^27–29^.

Polyesters have previously been used as core-forming blocks in the formation of well-defined 1D and 2D particles using living CDSA methods^30–33^. Such cores are inherently biocompatible, but also display hydrolytic biodegradation pathways in which the rate of degradation is controlled by their specific chemical structure^34^. The creation of particles with segmented cores that are derived from biocompatible and biodegradable polymers would lead to key advances through the generation of self-assembled nanoparticles in which the degradation properties and level of functionalization can be selectively manipulated.

In this article we demonstrate a versatile method to prepare a broad range of 2D segmented platelets with compositionally distinct cores. As a proof of concept, we show segmented 2D platelet micelles in which the core regions derived from polyesters with different hydrophobicity exhibit selective hydrolytic degradation. Our results should guide the future design and fabrication of spatially defined core–shell nanoparticles with diverse core compositions containing different functionalities.

## Results and discussion

We focused on the living CDSA of polylactone-based homopolymer and block copolymer blends to access precisely defined 2D nanostructures in which the dimensions can be controlled via the polymer-blend to seed-mass ratio. We first sought to demonstrate that different polylactone core-forming blocks could yield compositionally homogeneous platelets using this approach. Homopolymers were prepared by ring-opening polymerization of lactones of different ring size with varying numbers of carbon atoms (C_*x*_, *x* = 5–8 or 12), to form polylactones with different C_*x*_ spacers, namely poly(δ-valerolactone) (PVL, C_5_), poly(ε-caprolactone) (PCL, C_6_), poly(ζ-heptalactone) (PHL, C_7_), poly(η-octalactone) (POL, C_8_) and poly(λ-dodecanolactone) (PDDL, C_12_). BCPs containing segments of these materials as crystallizable, solvophobic core-forming blocks and poly(dimethylacrylamide) (PDMA) as a solvophilic corona-forming block were prepared by reversible addition-fragmentation chain-transfer (RAFT) polymerization (Supplementary Scheme 1, Supplementary Fig. 1 and Supplementary Table 1). All of the BCPs, PVL_80_-b-PDMA_295_, PCL_62_-b-PDMA_270_, PHL_50_-b-PDMA_217_, POL_55_-b-PDMA_280_ and PDDL_40_-b-PDMA_260_ (the subscripts refer to the number-average degree of polymerization), formed polydisperse cylinders by spontaneous nucleation in ethanol at 5 mg ml^−1^ when heated at 70 °C for 3 h and subsequently cooled to room temperature (25 °C). Near-uniform 1D crystalline seeds, ~60 nm in length and with low dispersity (*L*_w_/*L*_n_ of ~1.1), could be obtained for all BCP cylinders after sonication for 20 min at 0 °C (Supplementary Fig. 2).

A homopolymer/block copolymer blend (1:1 weight ratio, wt/wt), dissolved in chloroform, was used to grow 2D platelet particles in the presence of 1D seeds with the same core composition, colloidally dispersed in ethanol (Supplementary Fig. 3). With the exception of the PVL (C_5_) system, we were able to achieve well-defined platelets with controlled size for all BCP systems. Analysis of the resulting platelets by transmission electron microscopy (TEM) and atomic force microscopy (AFM) showed that, in each case, the 2D structure formed is highly regular in shape, and that their formation follows a living growth mechanism characterized by the linear relationship of the platelet area with the unimer-to-seed mass ratios (Supplementary Figs. 4–7). Both the homopolymer and BCP play critical roles in the formation of 2D platelets. In the absence of added homopolymer, growth occurs in 1D, and cylinders of controlled dimensions are generated (Supplementary Fig. 8). Without the addition of the BCP, growth does not occur in either 1D or 2D (Supplementary Fig. 8). In all of the subsequent work we used a mass ratio of 1:1 for homopolymer and BCP in the unimer blend to generate 2D platelets using the living CDSA method.

**Fig. 1:**
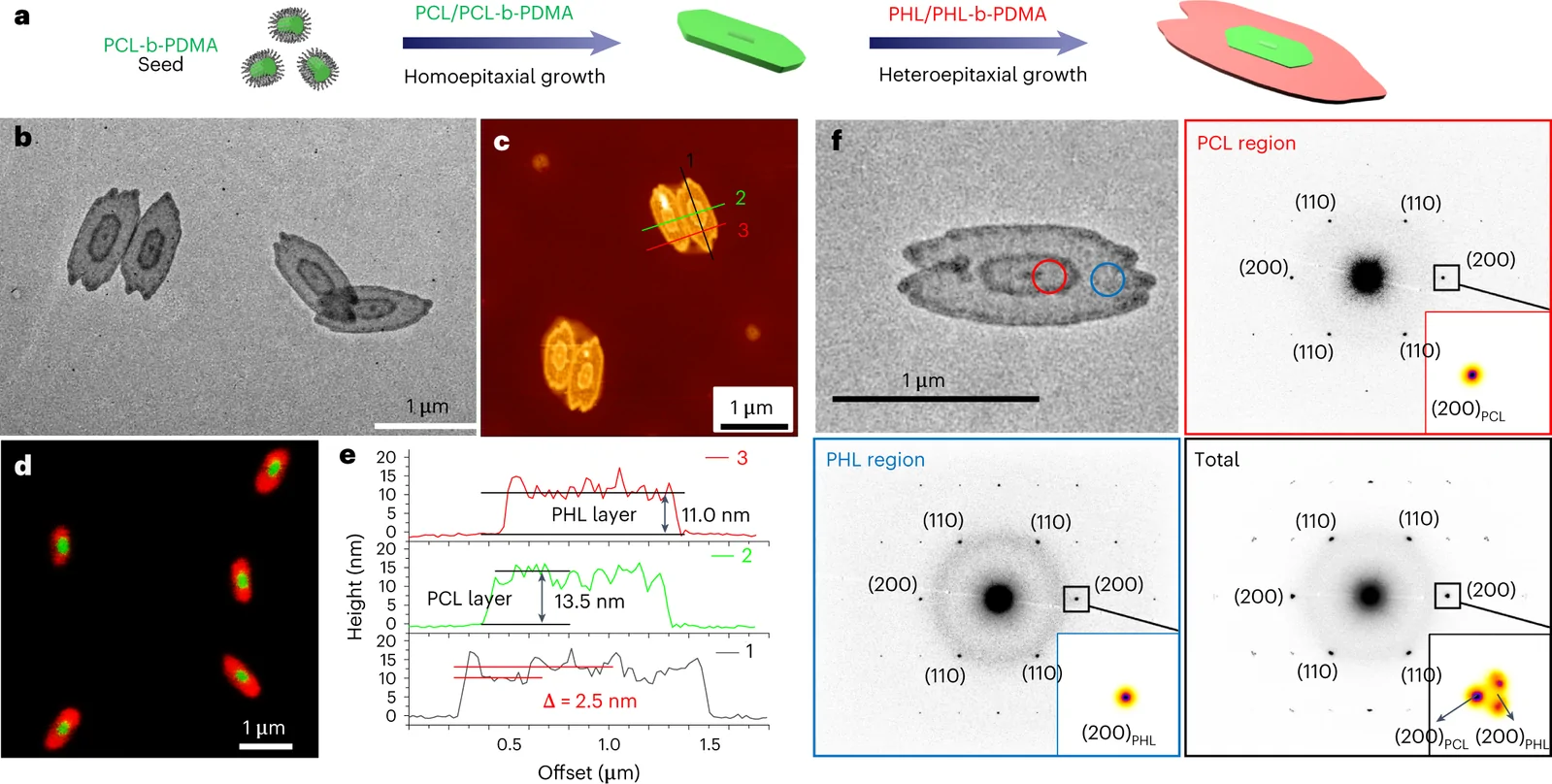
Uniform 2D platelets with segmented cores obtained by sequential growth of PCL (C_6_) and PHL (C_7_) homopolymer/block copolymer blends (1:1, wt/wt in CHCl_3_) from 1D PCL_62_-b-PDMA_270_ (C_6_) seeds colloidally dispersed in EtOH. **a**–**f**, Scheme for the formation of 2D PCL–PHL (C_6_–C_7_) block co-micelles (**a**), TEM image (**b**), AFM height image (**c**), STED image of BODIPY-dye-labelled platelet block co-micelles (**d**), height profiles of PCL–PHL block co-micelles corresponding to the numbered lines in **c** (**e**) and TEM image and SAED patterns for a PCL–PHL platelet block co-micelle (**f**). The PCL and PHL domains can be clearly distinguished from the TEM and AFM height images.

Next, to create core–shell nanoparticles with spatially and compositionally distinct cores, we investigated the seeded growth of different polylactone-containing polymer/block polymer blends by adding blend unimers of the other polylactones to PCL_62_-b-PDMA_270_ (C_6_) seeds. We initially investigated the growth of unimers of the PHL blend (C_7_) from a PCL_62_-b-PDMA_270_ (C_6_) seed in ethanol. Interestingly, this yielded more regular 2D platelets than those formed by spontaneous nucleation of a PHL blend (Supplementary Fig. 8). Moreover, the growth of a PHL core domain from 2D PCL platelet micelles could also be readily achieved with excellent control (Fig. 1). The two platelet regions of the diblock co-micelles (PCL–PHL, representing diblock co-micelles containing inner PCL (C_6_) and outer PHL (C_7_) compositions, the same as other block co-micelles) were clearly distinguishable from TEM and AFM height images, from which height profile analysis indicated that the height of the PHL region was ~2.5 nm lower than the PCL region. To provide further evidence that the micelles possessed a segmented structure, the added homopolymer was labelled with a red (PHL) or green (PCL) BODIPY dye (Supplementary Scheme 2). To this end, by sequential addition of these labelled blend unimers to a PCL_62_-b-PDMA_270_ seed, concentric 2D platelets with compositionally distinct cores and spatially defined fluorescent coronal regions were obtained, as shown by stimulated emission depletion (STED) microscopy (Fig. 1d).

To obtain insight into the mechanism for the successful creation of 2D core–shell particles with spatially and compositionally distinct cores, characterization of the relevant crystal lattices was performed. Heteroepitaxial growth with crystallizable polymers is limited to cases where the lattice mismatch does not exceed 15%^35^. Wide-angle X-ray diffraction (WAXD) analysis of powder samples revealed that all of the polylactone samples have very similar (110) crystalline peaks (Supplementary Fig. 9 and Supplementary Table 2). In the case of a PHL (C_7_) core, however, a slight difference in the (200) crystalline peak in comparison with the other samples in the series is apparent, but even this only corresponds to a difference of ~2% (*d* = 0.365 nm versus 0.371 nm) (Supplementary Fig. 9 and Supplementary Table 2). These results therefore indicate that a heteroepitaxial growth mechanism is potentially possible for the polylactones according to the lattice-matching criterion. We then used high-resolution selected-area electron diffraction (SAED) to analyse the crystal structures of the different domains of platelets with spatially and compositionally distinct polylactone cores for the case involving the largest lattice-spacing difference for the components. The SAED pattern obtained from a 2D PHL (C_7_) platelet grown from a 1D PCL_62_-b-PDMA_270_ (C_6_) seed displays an identical *d*-spacing of (200) to that for a compositionally homogeneous PHL platelet (*d* = 0.365 nm), but differs from that for a compositionally pure PCL platelet (*d* = 0.371 nm) (Supplementary Fig. 9). Moreover, analysis of the spatially distinct regions of segmented 2D platelets with different PCL/PHL (C_6_/C_7_) domains confirmed that the crystalline structure of PCL-based and PHL-based regions is also different (*d* = 0.371 nm for the PCL region but 0.365 nm for the PHL region). This difference could be further verified from the overall SAED pattern for the complete platelet, which revealed separation of the PCL and PHL (200) diffraction spots (Fig. 1f), corresponding to *d*-spacings of 0.371 and 0.365 nm, respectively. These results indicate that, if a heteroepitaxial growth mechanism is operational, the crystal lattice of the newly grown PHL segment relaxes from that imposed by the underlying PCL substrate. Another mechanistic possibility involves an initial step in which secondary nucleation of crystalline PHL occurs on the PCL surface at the growth frontier. Subsequent homoepitaxial growth of PHL from the deposited crystals would form an outer platelet region with the characteristic PHL crystal lattice. The results from our diffraction studies contrast with those in previously reported cases of heteroepitaxial growth involving PFS- and PFG-based BCPs, where the core lattice structure of the underlying PFS substrate at the micelle seed termini was imposed throughout the newly grown PFG region^28,29^. However, the results are consistent with those for core–shell platelets formed by heteroepitaxial growth of different collagen-mimetic peptide-based BCPs^14^ and the formation of dendritic micelles, where a branching mechanism that involves secondary nucleation has been invoked^29^.

**Fig. 2:**
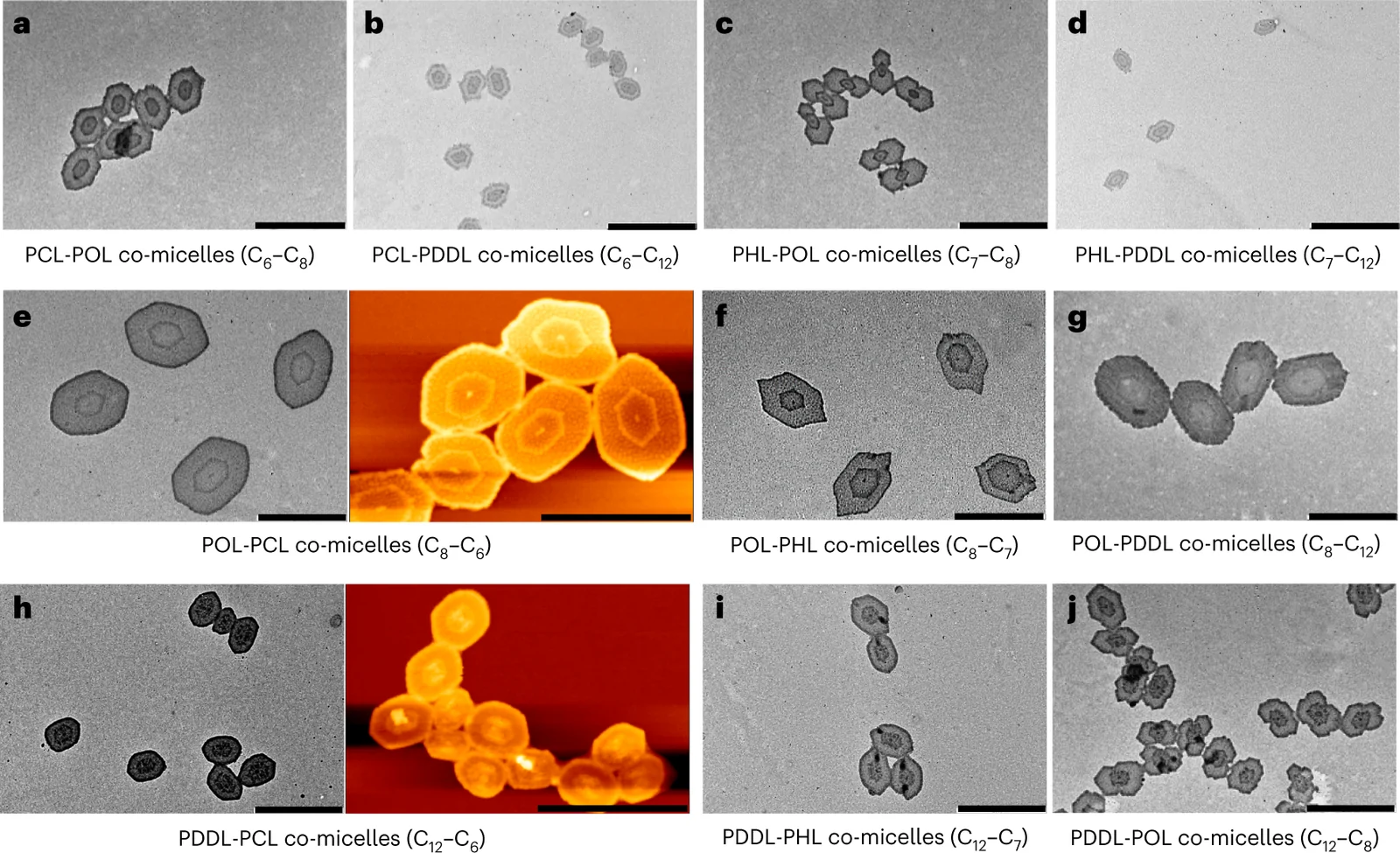
Two-dimensional platelet block co-micelles formed from four different polylactones. **a**–**j**, TEM images of PCL–POL (C_6_–C_8_) (**a**), PCL–PDDL (C_6_–C_12_) (**b**), PHL–POL (C_7_–C_8_) (**c**), PHL–PDDL (C_7_–C_12_) (**d**), POL–PCL (C_8_–C_6_) (**e**), POL–PHL (C_8_–C_7_) (**f**), POL–PDDL (C_8_–C_12_) (**g**), PDDL–PCL (C_12_–C_6_) (**h**), PDDL–PHL (C_12_–C_7_) (**i**) and PDDL–POL (C_12_–C_8_) (**j**) block co-micelles prepared by a sequential seeded growth approach in ethanol. The right-hand images of POL–PCL (C_8_–C_6_) (**e**) and PDDL–PCL (C_12_–C_6_) (**h**) are AFM height images. The different sizes of the block co-micelles obtained in each case are due to the different molecular weights of the crystallizable homopolymers and block copolymers. Scale bars = 2,000 nm.

To extend the use of the seeded growth approach to a polylactone core that has notably different hydrophobicity, we examined the living CDSA of polylactone blends containing longer (POL, C_8_ and PDDL, C_12_) or shorter carbon chains (PVL, C_5_) from 1D seeds and 2D platelet micelles with a crystalline PCL core (C_6_). We hypothesized that, based on previous polylactone degradation studies, the different hydrophobicity would lead to different degradation rates within the same particle^36^, thereby enabling access to 2D nanostructures with cores that were capable of programmed degradation. Interestingly, successful growth was achieved for the cases of the longer-chain polylactones, as evidenced by the formation of well-defined PCL–POL (C_6_–C_8_) and PCL–PDDL (C_6_–C_12_) segmented platelets with controlled dimensions upon the addition of POL or PDDL homopolymer and BCP blend unimers to either 2D platelets or 1D PCL_62_-b-PDMA_270_ seed micelles (Fig. 2a,b and Supplementary Figs. 10 and 11). However, attempts to establish the growth of blends based on PVL (C_5_) from other polylactone-based 1D seed micelles or 2D platelets only yielded poorly defined nanoparticles (Supplementary Fig. 12). To further explore the versatility and scope of this approach, we investigated growth of blends from 2D platelets with crystalline polylactone cores based on C_7_, C_8_ and C_12_ spacers. Impressively, this was successful for all of the polylactones in the series, allowing us to grow a wide range of segmented 2D platelets with spatially and chemically distinct polylactone cores (Fig. 2). In addition, controlled growth of other polylactone cores based on C_6_, C_7_, C_8_ and C_12_ spacers from 1D PVL (C_5_) seeds also yielded well-developed segmented platelets (Supplementary Fig. 13). The exceptions were associated with the attempted use of PCL (C_6_) blend unimer for growth from a PHL (C_7_) seed core or a PVL (C_5_) blend unimer from any of the polylactone seeds (Supplementary Figs. 10 and 12).

To provide further insight into the limitations of the seeded growth approach, the unsuccessful case involving blends based on a PCL (C_6_) core-forming block and seed micelles and platelets based on PHL (C_7_) was examined in more detail. It is possible that, in this case, growth was not observed at ambient temperature as a consequence of discrepancies in core thickness between the newly deposited PCL nucleus and PHL seed^37^. In our experiments, spontaneous nucleation of the PCL components of the blend dominated at elevated temperature (40 °C) to form large polydisperse platelets (Supplementary Fig. 14). In contrast, at lower temperatures (−25 to 4 °C), where any thickness discrepancy should be reduced, segmented platelets were successfully formed, but these were not well-defined (Supplementary Fig. 14). To overcome this limitation, we attempted to change the PCL microstructure in the polymer components through copolymerization with a PHL monomer. We therefore prepared two PCL/PHL (C_6_/C_7_) copolymers, one a BCP obtained by sequential monomer addition, P(CL_32_-b-HL_33_), and one a statistical copolymer through copolymerization, P(CL_32_-co-HL_28_). We then investigated their potential growth from 1D PHL_50_-b-PMDA_217_ (C_7_) seeds when used in homopolymer/block copolymer blends. Notably, blended unimers of P(CL_32_-b-HL_33_)/P(CL_32_-b-HL_33_)-b-PDMA_178_, which possessed a block PCL/PHL (C_6_/C_7_) microstructure, grew from PHL (C_7_) seed micelles and platelets (Supplementary Fig. 15) to form well-defined platelet block co-micelles. This is probably the result of the increased similarity between the chemistries of the unimer components and seeds, improving their compatibility for sequential growth. In contrast, unimers of a P(CL_32_-co-HL_28_)/P(CL_32_-co-HL_28_)-b-PDMA_268_ blend containing the statistical copolymer P(CL_32_-co-HL_28_) (C_6_/C_7_) core did not allow effective 2D living growth. This is most likely as a consequence of reduced crystallizability and enhanced solubility of the statistical random copolymer structure (Supplementary Fig. 15).

To address the limitation that the PVL (C_5_) blend could not be grown from any other polylactone-based seeds, we attempted to copolymerize VL (C_5_) with the CL (C_6_) monomer. In this case, the statistical copolymerization of VL and CL monomers yielded a blocky PVL/PCL copolymer, P(VL_30_-co-CL_35_), as evidenced by ^13^C NMR spectroscopic analysis (Supplementary Fig. 16). Differential scanning calorimetry (DSC) and WAXD results confirmed the isomorphic crystallization of P(VL_30_-co-CL_35_) (Supplementary Fig. 16) arising from the co-crystallization of VL and CL units^38^. This copolymer was then used to generate P(VL_30_-co-CL_35_)-b-PDMA_265_ by RAFT polymerization. To confirm that this BCP underwent living CDSA, P(VL_30_-co-CL_35_)-b-PDMA_80_ was used to prepare 1D seed micelles by the previously described method (Supplementary Fig. 17). Addition of P(VL_30_-co-CL_35_)/P(VL_30_-co-CL_35_)-b-PDMA_265_ blend unimer to the seed micelles formed from P(VL_30_-co-CL_35_)-b-PDMA_80_ yielded 2D platelets; however, the morphologies were not as well developed as those described for the other polylactone-based systems (Supplementary Fig. 18). The 2D platelets of P(VL_30_-co-CL_35_)/P(VL_30_-co-CL_35_)-b-PDMA_265_ formed by seeded growth appear to possess a less-ordered core based on the relative breadth of the WAXD peaks (Supplementary Fig. 18) and the reduced crystallization/melting temperatures for P(VL_30_-co-CL_35_) compared to PCL from DSC analysis (Supplementary Fig. 16) and spontaneous nucleation experiments (Supplementary Fig. 17).

**Fig. 3:**
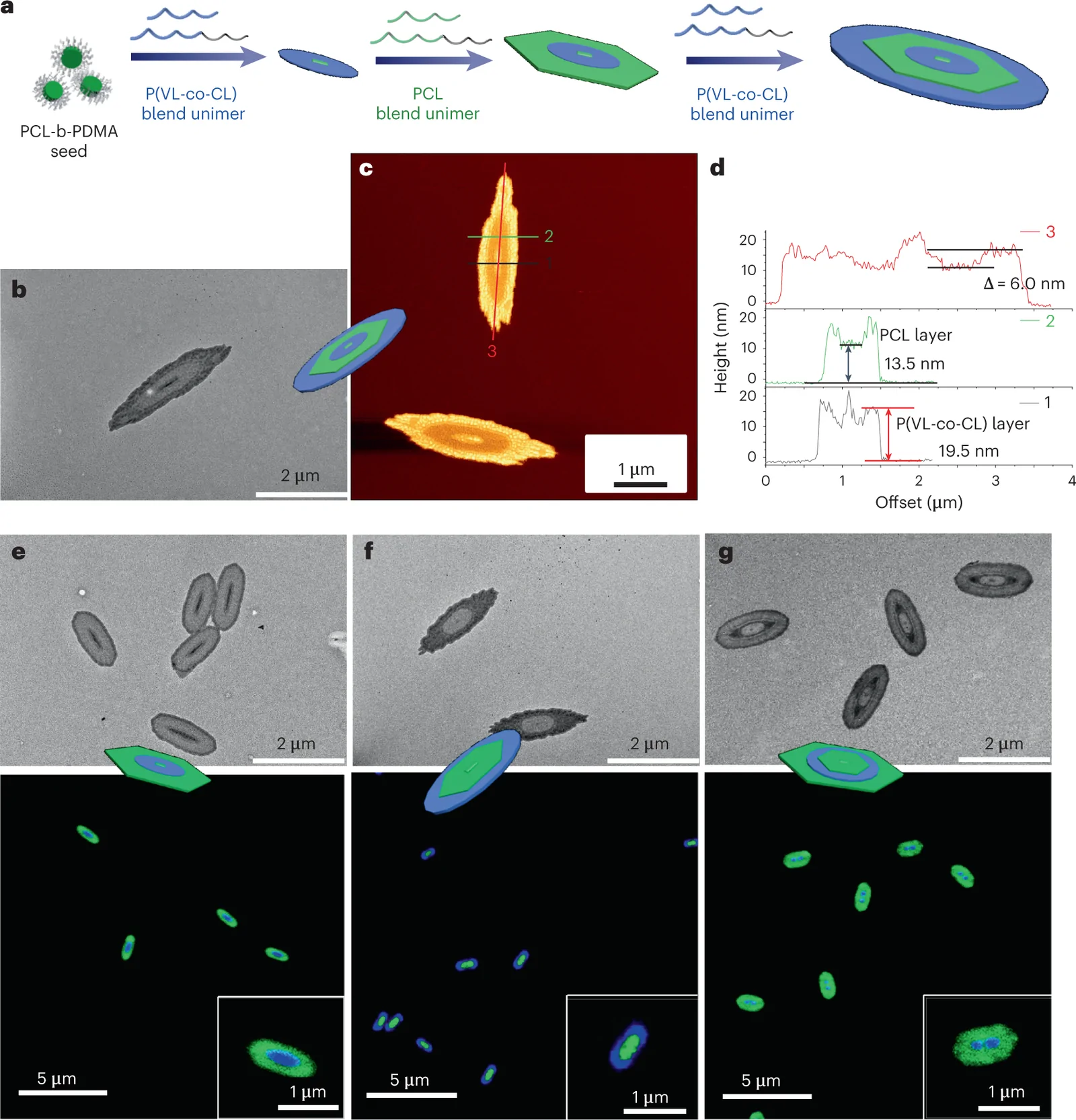
Uniform 2D AB di- and ABA triblock segmented platelets with spatially distinct core chemistries prepared by seeded growth of P(VL-co-CL)-based blend unimer and PCL-based blend unimer using 1D PCL_62_-b-PDMA_270_ seeds. **a**–**d**, Schematic representation of the formation of triblock co-micelles from a PCL_62_-b-PDMA_270_ seed (**a**), TEM image (**b**), AFM height image (**c**) and corresponding height information of the lines indicated in **c** (**d**) for the triblock co-micelle. **e**–**g**, TEM and corresponding STED images of 2D platelets labelled with BODIPY dyes: diblock co-micelles of P(VL-co-CL)-PCL prepared from sequential addition of P(VL-co-CL) and PCL blend unimer into 1D PCL_62_-b-PDMA_270_ seeds (**e**), diblock co-micelle of PCL-P(VL-co-CL) prepared from sequential addition of PCL and P(VL-co-CL) blend unimer into 1D PCL_62_-b-PDMA_270_ seeds (**f**) and triblock co-micelles of PCL-P(VL-co-CL)-PCL prepared from sequential addition of PCL, P(VL-co-CL) and PCL blend unimer into 1D PCL seeds (**g**). The PCL region is labelled with green dye, and the P(VL-co-CL) region with blue dye.

Interestingly, the P(VL_30_-co-CL_35_)-b-PDMA_80_ (C_5_/C_6_) seed micelles could be used for the subsequent growth of other homopolymer/BCP blends based on PCL (C_6_), PHL(C_7_), POL (C_8_) and PDDL (C_12_) as the core-forming block. Well-defined platelets were obtained in all cases (Supplementary Fig. 19). Furthermore, addition of P(VL_30_-co-CL_35_)/P(VL_30_-co-CL_35_)-b-PDMA_265_ (C_5_/C_6_) blend unimer to PCL_62_-b-PDMA_270_ (C_6_) seeds led to the formation of 2D platelets with well-defined morphology and tunable dimensions that were dependent on the unimer-to-seed ratio (Fig. 3 and Supplementary Fig. 20). The resulting 2D platelets were then used as seed precursors for the subsequent growth of a second PCL-based platelet region and then a third region based on P(VL-co-CL) by the sequential addition of unimers of the corresponding blends. This yielded 2D platelet AB diblock and ABA triblock co-micelles in which the different concentric regions were easily distinguishable by TEM and AFM imaging (Fig. 3b–d). This approach could be further extended to afford well-defined ABABAB hexablock co-micelles with uniform size by the further sequential addition of unimers of PCL-, P(VL-co-CL)- and PCL-based blend unimers (Supplementary Fig. 21). To provide further evidence for the multiblock nature of the platelet micelles, in one case the different spatially defined segments were labelled with BODIPY fluorescent dyes (P(VL-co-CL) blue, PCL green) followed by analysis by STED microscopy. This enabled clear visualization of the distinct concentric regions of the segmented platelets (Fig. 3e–g).

**Fig. 4:**
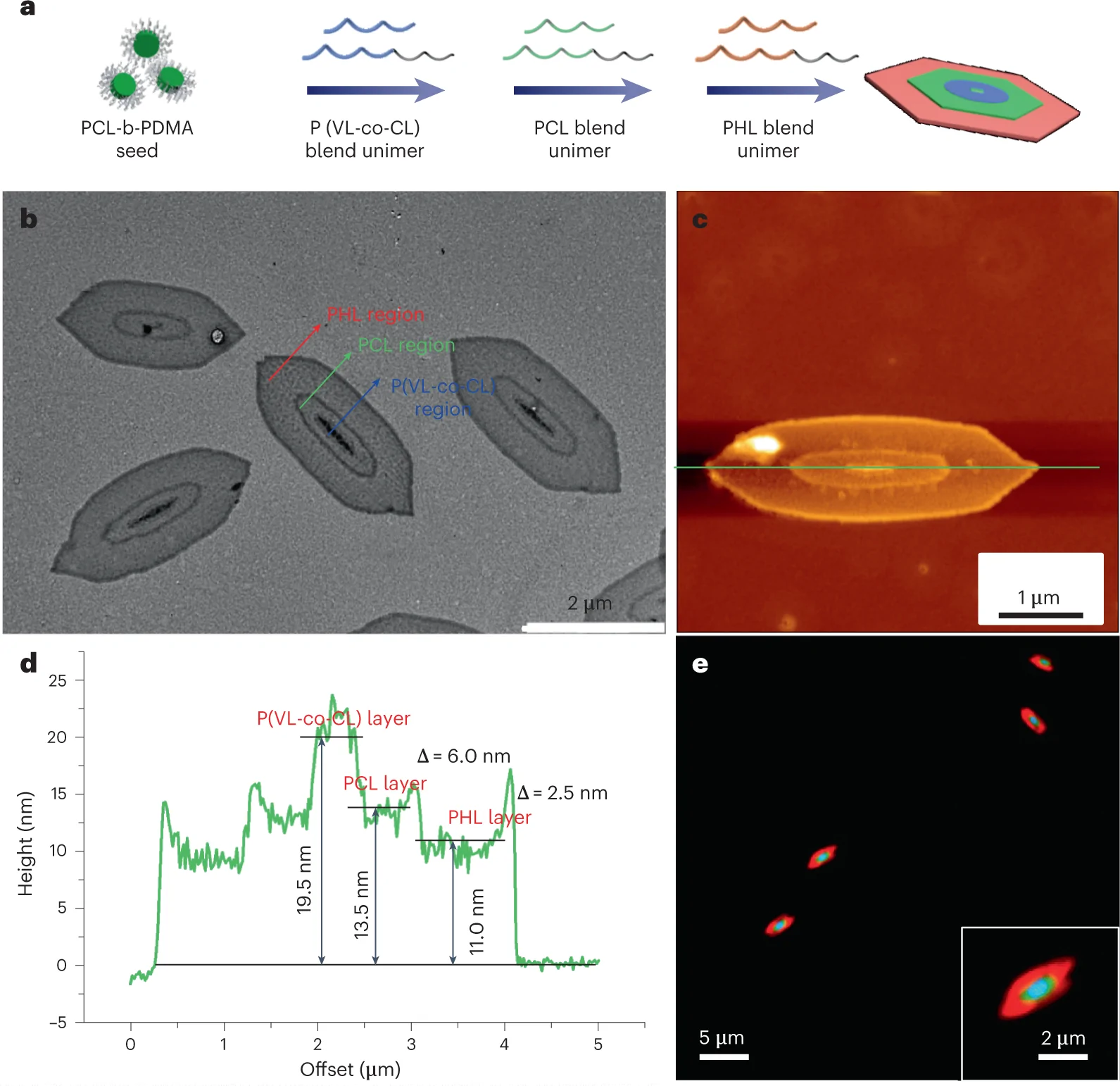
Formation of 2D ABC triblock segmented platelets with distinct core chemistries by seeded growth. **a**, Scheme of uniform 2D platelet block co-micelles prepared by sequential seeded growth of P(VL-co-CL), PCL and PHL blend unimers from 1D PCL_62_-b-PDMA_270_ seeds. **b**–**d**, TEM image (**b**), AFM height image (**c**) and corresponding height profile (**d**) of triblock co-micelles in **c**. **e**, Corresponding STED images of BODIPY-dye-labelled 2D triblock co-micelles; P(VL-co-CL) is labelled in blue, PCL in green and PHL in red.

To further demonstrate the scope and expand the complexity of the 2D platelets with segmented cores formed through this seeded growth process, an ABC triblock co-micelle platelet that contained P(VL-co-CL) (C_5_/C_6_), PCL (C_6_) and PHL (C_7_) cores was prepared by sequential addition of the three different blend unimers to 1D PCL_62_-b-PDMA_270_ (C_6_) seeds. This afforded triblock co-micelle platelets with uniform sizes, as confirmed by TEM and AFM analysis (Fig. 4). The AFM height profile of the platelets showed that the three different layers all had different heights (Fig. 4d), consistent with triblock co-micelle formation. This was further confirmed using dye-labelled P(VL-co-CL), PCL and PHL copolymers, as evidenced by the fluorescent concentric platelet multiblock co-micelles with readily distinguishable regions imaged by STED (Fig. 4e).

**Fig. 5:**
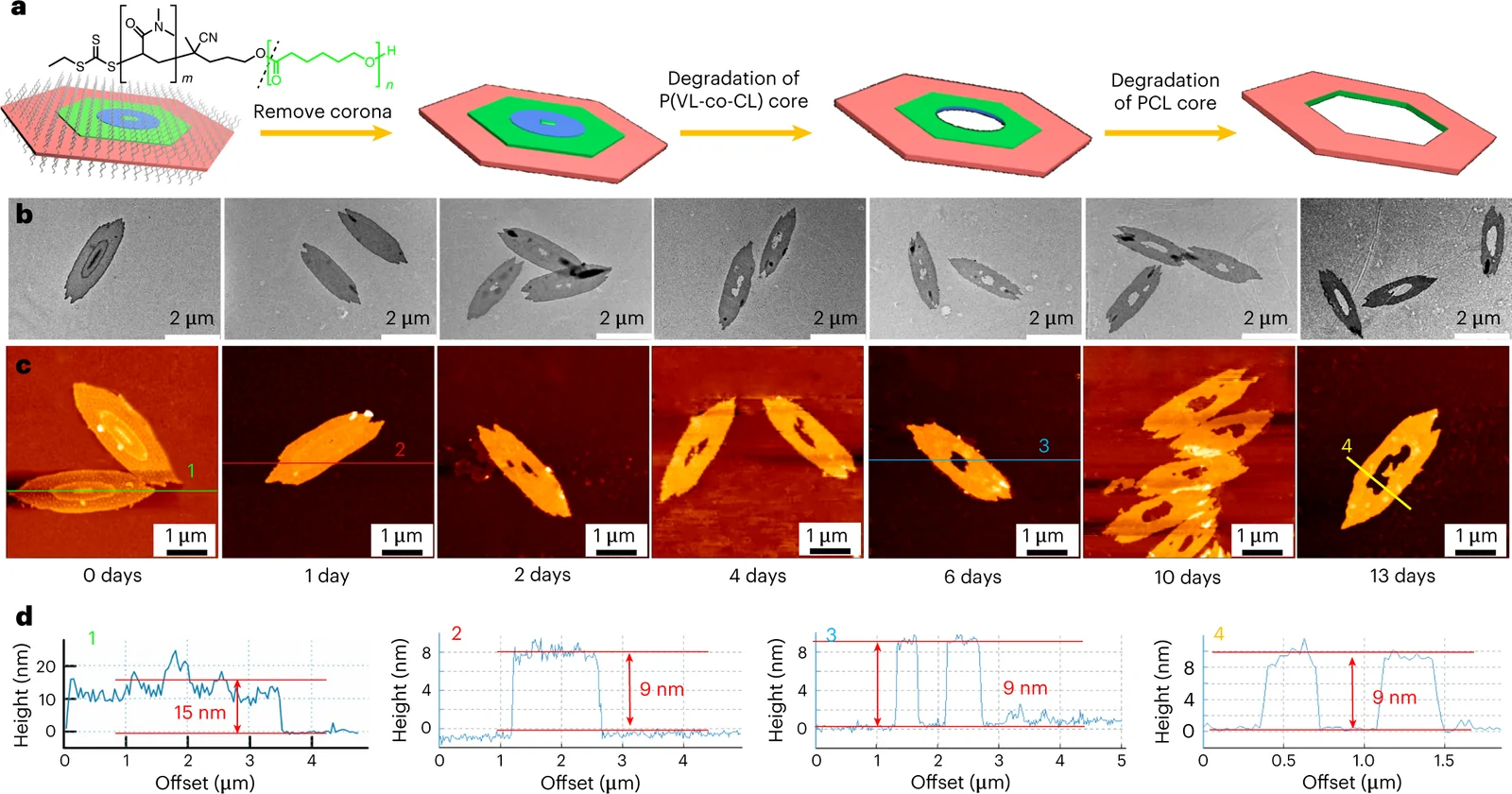
Time-resolved degradation for the 2D ABC triblock segmented platelets with a P(VL-co-CL)-PCL-PHL core in an aqueous 1 M KOH solution. **a**–**d**, Schematic illustration of the degradation process, where wavy grey lines represents the corona block of PDMA, and the blue, green and red colours correspond to P(VL-co-CL), PCL and PHL core components, respectively (**a**), the TEM morphologies (**b**) and corresponding AFM height images (**c**), and the height profiles of the lines indicated in **c** (labelled 1, 2, 3 and 4) (**d**) of the triblock co-micelles at different degradation times.

To exploit the different degradation profiles of the core-forming polylactones within the multiblock co-micelle platelets, we investigated their selective degradation in an aqueous basic environment (Fig. 5). First, ABC 2D platelet triblock co-micelles with P(VL-co-CL) (A, inner), PCL (B, central) and PHL (C, outer) core domains were transferred into water (from EtOH) by dialysis, with no change in size or morphology (Supplementary Fig. 22). However, after degradation for one day, the platelet surface appeared to become smooth (Fig. 5b,c, 1 day), most likely as a consequence of the corona block on the platelet surface being removed by hydrolysis of the ester bond at the exposed interface between core and corona. This was evidenced by a reduction in the AFM height profile from an average of ~15 nm to ~9 nm (Fig. 5d, 1 and 2). After two days, the inner core domain, P(VL-co-CL) (A, which is the least hydrophobic), was observed to degrade, appearing to completely disappear after four days (Fig. 5b,c, 4 days and Fig. 5d, 3). During this time, AFM and TEM analysis strongly suggested that the central and outer core domain layers of PCL (B) and PHL (C) remained intact. However, after 10–13 days under these conditions, the core void was greatly enlarged, which was also consistent with PCL degradation (Fig. 5b,c, 13 days and Fig. 5d, 4). Through the careful choice of degradation conditions, the selective removal of the central (rather than inner) core domains of ABA triblock co-micelles with PCL (A, inner and outer) and P(VL-co-CL) (B, central) core regions could also be achieved (Supplementary Fig. 23). Unlike the previous fabrication of hollow platelet structures, which requires several synthetic steps, including crosslinking of the peripheral section and dissolution of the central crosslink-free region^20,39^, this approach, involving selective degradation, represents a more efficient route that avoids time-consuming post-modification steps.

## Conclusions

We have demonstrated that a seeded growth approach can be applied to a range of different crystallizable polymer blends based on different polylactone-based core-forming homopolymer/block copolymer combinations. This allows access to 2D platelet micelles with segmented and distinct core chemistries and with precise control over dimensions. This approach is robust and versatile, and represents a key advance in the development of anisotropic and chemically complex polymer nanostructures. The degradation-rate differences between the polylactone cores can be exploited to create particles in which selective degradation can occur in a spatiotemporally controlled manner, which could enable a range of applications, especially in controlled release and cargo delivery. The distinct responsive behaviour in particles that can be ‘turned on’ under programmed conditions may offer opportunities to create micrometre-scale carriers that are capable of long-term programmed drug release as therapeutic delivery vehicles of the future. Moreover, we have demonstrated that three key factors—crystallization kinetics, crystallizability and core compatibility—are of major importance with respect to regulation of the heteroepitaxial growth behaviour of crystallizable homopolymer/block copolymer blends. The growth method we describe offers a potentially general approach to 2D platelet micelles with distinct programmable core chemistries. For example, an extension of the method to crystallizable *π*-conjugated polymers with distinct bandgaps as core-forming blocks would allow access to 2D assemblies with potential applications in the field of nanoelectronic heterojunctions.

## Methods

### Materials

We distilled CL, VL and DDL under vacuum over calcium hydride before being introduced into a glovebox and used. Diphenyl phosphate (DPP) was recrystallized from dried CHCl_3_/hexane (3:1) and dried over P_2_O_5_. 1,4-Dioxane and DMA were purified by passing through basic alumina before use. 2,2′-Azobis(2-methylpropionitrile) (AIBN) was recrystallized twice from methanol and stored in the dark at 4 °C. The dual-functional chain transfer agent (CTA), 4-cyano-4-(((ethylthio)carbonothioyl)thio)pentanoic acid (CEPA) and PCL-b-PDMA were prepared according to our previous literature^31^. The catalyst magnesium 2,6-di-*tert*-butyl-4-methylphenoxide (Mg(BHT)_2_(THF)_2_) was synthesized according to a previous paper^40^. The BODIPY dyes were purchased from Lumiprobe and used as received.

### Characterizations

^1^H NMR and ^13^C NMR spectra were recorded at 400 MHz on a Bruker DPX-400 spectrometer in CDCl_3_ solvent. Gel permeation chromatography (GPC) measurements were performed on a Varian 390-LC-Multi detector suite system fitted with refractive index (RI) and ultraviolet (UV) detectors (*λ* = 309 nm) equipped with a PLGel 3-μm (50 × 7.5 mm) guard column and two PLGel 5-μm (300 × 7.5 mm) mixed-D columns using dimethyl formamide or CHCl_3_ with 5 mM NH_4_BF_4_ at 50 °C as the eluent at a flow rate of 1.0 ml min^−1^. GPC data were calibrated against poly(methyl methacrylate) (PMMA) standards and analysed using Cirrus v3.3 software. Samples for TEM analysis were prepared by drop-casting 5 µl of micellar solution onto a carbon-coated copper grid placed on filter paper. Cylinder samples were stained with 1 wt% uranyl acetate aqueous solution. Imaging for samples was performed on a JEOL 1400 transmission electron microscope operating at 80 kV. Samples for AFM analysis were prepared by drop-casting 10 µl of micellar solution onto the silicon wafer. Imaging and analysis were performed on an Asylum Research MFP3D-SA atomic force microscope in QI mode. Dye-labelled platelet images were obtained using digital detectors with observation windows of 605–700 nm (R), 500–570 nm (G) and 415–470 (B). STED microscopy was carried out on a Leica TCS SP8 STED 3x microscope running LAS X acquisition software using a ×100 HC PL APO CS2 oil immersion objective (NA 1.4). Fluorescence depletion was accomplished using a continuous-wave 590-nm STED laser (1.5-W nominal power, 60%). All images were acquired at a scan speed of 200 Hz in monodirectional scan mode with resolution of 1024*1024 pixeks and a pixel size of *ca*. 80 nm^2^. The fluorescence signal was detected by a hybrid detector (HyD, Standard mode) after passing through an acousto-optical beamsplitter. For imaging of the multiblock co-micelles, the output power of each laser was varied until the fluorescence of all blocks could be observed at approximately equal brightness. WAXD patterns were obtained with an X’Pert PRO instrument (PANalytical BV) at room temperature using Ni-filtered Cu K*α* radiation with a wavelength of 1.54 Å as the X-ray source.

### Synthesis of ζ-heptalactone and η-octalactone monomers

ζ-heptalactone (HL) and η-octalactone (OL) monomers were prepared from Baeyer−Villiger oxidations of corresponding cycloheptanone and cyclooctanone, respectively, as reported elsewhere^41^. Typically, the appropriate cycloheptanone (223 mmol) and *m*-chloroperbenzoic acid (275 mmol) were mixed in CH_2_Cl_2_ (250 ml). The suspension was heated under reflux for three days. The reaction mixture was cooled in an ice bath, and the solids were filtered over Celite and washed with CH_2_Cl_2_ (2 × 50 ml). The filtrate was then washed with 10% Na_2_S_2_O_3_ solution (2 × 200 ml), saturated Na_2_CO_3_ solution (2 × 200 ml) and saturated NaCl solution (1 × 200 ml). The organic layer was dried with MgSO_4_, filtered, and evaporated in vacuo. The resulting liquid was distilled for purification to yield HL monomer (20.0 g, 156 mmol, in yield of 70%). ^1^H NMR (CDCl_3_): *δ*/ppm 4.28 (t, 2H, C*H*_*2*_O), 2.48 (t, 2H, C*H*_*2*_C = O), 1.75 (m, 4H, C*H*_*2*_), 1.52 (m, 4H, C*H*_*2*_). The synthesis of OL was the same as that of HL (19.6 g, 138.3 mmol, in yield of 62%). ^1^H NMR (400 MHz, CDCl_3_): *δ*/ppm 4.25 (t, 2H, C*H*_*2*_O), 2.25 (t, 2H, C*H*_*2*_C = O), 1.70 (m, 6H, C*H*_*2*_), 1.40 (m, 4H, C*H*_*2*_).

### Synthesis of PHL, POL and PDDL via ring-opening polymerization

The ring-opening polymerization method used to prepare longer-carbon-chain polylactones (PHL, POL and PDDL) is described in our reported paper^42^. The synthetic routine is shown in Supplementary Scheme 1. Typically, in an inert nitrogen glovebox, catalyst Mg(BHT)_2_(THF)_2_ (8.0 mg, 13 μmol), CTA (6.5 mg, 26 μmol), HL (0.2 g, 1.56 mmol) and dry toluene (1.0 ml) were charged into an ampoule. The ampoule was sealed for 1 h. The reaction was quenched by the addition of acidified (5% HCl) methanol. Chloroform was added to dissolve solids, and the polymer was precipitated in excess methanol three times. The synthesis method for POL and PDDL homopolymer was the same as that for PHL.

PHL_50_: ^1^H NMR (400 MHz, CDCl_3_) *δ*/ppm: 4.06 (t, C*H*_*2*_O), 3.65 (2H, C*H*_*2*_OCO), 2.30 (t, OCOC*H*_*2*_), 1.73–1.33 (m, C*H*_*2*_C*H*_*2*_C*H*_*2*_C*H*_*2*_); GPC (CHCl_3_, PMMA standard): Number average molecular weight, *M*_n_ = 8.3 kg mol^−1^,dispersity, *Đ*_M_ = 1.17.

POL_55_: ^1^H NMR (400 MHz, CDCl_3_) *δ*/ppm: 4.06 (t, C*H*_*2*_O), 3.65 (2H, C*H*_*2*_OCO), 2.30 (t, OCOC*H*_*2*_), 1.73–1.33 (m, C*H*_*2*_C*H*_*2*_C*H*_*2*_C*H*_*2*_ C*H*_*2*_); GPC (CHCl_3_, PMMA standard): *M*_n_ = 11.5 kg mol^−1^, *Đ*_M_ = 1.34.

PDDL_40:_^1^H NMR (400 MHz, CDCl_3_) *δ*/ppm: 4.06 (t, C*H*_*2*_O), 3.65 (2H, C*H*_*2*_OCO), 2.30 (t, OCOC*H*_*2*_), 1.73–1.33 (m, C*H*_*2*_C*H*_*2*_C*H*_*2*_C*H*_*2*_ C*H*_*2*_C*H*_*2*_C*H*_*2*_C*H*_*2*_C*H*_*2*_); GPC (CHCl_3_, PMMA standard): *M*_n_ = 12.8 kg mol^−1^, *Đ*_M_ = 2.21.

### Synthesis of PVL and PCL via ring-opening polymerization

Typically, the synthetic process for the PCL homopolymer was as follows. In a nitrogen-filled glovebox, solutions of DPP (42 mg, 0.18 mmol) and CTA (45 mg, 0.18 mmol) in dry toluene (10 ml) were added to CL (1.44 g, 12.6 mmol). After stirring for 8 h at room temperature, the solution was removed from the glovebox, precipitated three times into ice-cold methanol, and collected by centrifugation. The synthetic process for PVL was the same as for PCL. The DSC cooling and heating scans of all the homopolymers are shown in Supplementary Fig. 26.

PCL_62_: ^1^H NMR (400 MHz, CDCl_3_) *δ*/ppm: 4.06 (t, C*H*_*2*_O), 3.65 (2H, C*H*_*2*_OCO), 2.30 (t, OCOC*H*_*2*_), 1.73–1.33 (m, C*H*_*2*_C*H*_*2*_C*H*_*2*_); GPC (CHCl_3_, PMMA standard): *M*_n_ = 12.3 kg mol^−1^, *Đ*_M_ = 1.10.

PVL_80_: ^1^H NMR (400 MHz, CDCl_3_) *δ*/ppm: 4.06 (t, C*H*_*2*_O), 3.65 (2H, C*H*_*2*_OCO), 2.30 (t, OCOC*H*_*2*_), 1.69–1.66 (m, C*H*_*2*_C*H*_*2*_); GPC (CHCl_3_, PMMA standard): *M*_n_ = 9.8 kg mol^−1^, *Đ*_M_ = 1.22.

### Synthesis of P(VL-co-CL) and P(CL-co-HL) via ring-opening polymerization

Typically, in an inert nitrogen glovebox, catalyst Mg(BHT)_2_(THF)_2_ (8.0 mg, 13 μmol), CTA (6.5 mg, 26 μmol), HL (0.116 g, 0.91 mmol), CL (0.104 g, 0.91 mmol) and dry toluene (1.0 ml) were charged into an ampoule. The ampoule was sealed for 1 h. The reaction was quenched by the addition of acidified (5% HCl) methanol. Chloroform was added to dissolve solids and the copolymer was precipitated in excess methanol three times, yielding the P(CL-co-HL) copolymer core. The synthesis process for P(VL-co-CL) core was completed in a similar way by using DPP as a catalyst instead of Mg(BHT)_2_(THF)_2_.

P(VL_30_-co-CL_35_): ^1^H NMR (400 MHz, CDCl_3_) *δ*/ppm: 4.06 (t, C*H*_*2*_O), 3.65 (2H, C*H*_*2*_OCO), 2.30 (t, OCOC*H*_*2*_), 1.73–1.33 (m, C*H*_*2*_C*H*_*2*_C*H*_*2*_ from CL; C*H*_*2*_C*H*_*2*_ from VL); ^13^C NMR (100 MHz, CDCl_3_) *δ*/ppm: 173.5 (CL, O*C*OCH_2_), 173.2 (VL, O*C*OCH_2_), 64.2 (CL*-VL, O*C*H_2_), 64.1 (CL*-CL, O*C*H_2_), 63.9 (VL*-VL, O*C*H_2_), 63.8 (VL*-CL, O*C*H_2_), 34.1 (CL, OCO*C*H_2_), 33.7 (VL, OCO*C*H_2_), 28.3 (CL, OCH_2_*C*H_2_), 28.0 (VL, OCH_2_*C*H_2_), 25.5 (CL, OCOCH_2_*C*H_2_), 24.5 (CL, OCOCH_2_CH_2_*C*H_2_), 21.4 (VL, OCOCH_2_*C*H_2_); GPC (CHCl_3_, PMMA standard): *M*_n_ = 11.8 kg mol^−1^, *Đ*_M_ = 1.17.

P(CL_32_-co-HL_28_): ^1^H NMR (400 MHz, CDCl_3_) *δ*/ppm: 4.06 (t, C*H*_*2*_O), 3.65 (2H, C*H*_*2*_OCO), 2.30 (t, OCOC*H*_*2*_), 1.73–1.33 (m, C*H*_*2*_C*H*_*2*_C*H*_*2*_ from CL; C*H*_*2*_C*H*_*2*_C*H*_*2*_C*H*_*2*_ from HL); 173.77 (HL*-HL, O*C*OCH_2_), 173,73 (HL*-CL, O*C*OCH_2_), 173.59 (CL*-HL, O*C*OCH_2_), 173.56 (CL*-CL, O*C*OCH_2_), 64.32 (HL*-CL, O*C*H_2_), 64.28 (HL*-HL, O*C*H_2_), 64.16(CL*-CL, O*C*H_2_), 64.12 (CL*-CL, O*C*H_2_), 34.21 (HL*-HL, OCO*C*H_2_), 34.19 (HL*-CL, OCO*C*H_2_), 34.15 (CL*-HL, OCO*C*H_2_), 34.14 (CL*-CL, OCO*C*H_2_), 28.78 (HL, OCH_2_*C*H_2_), 28.49 (CL*-HL, OCH_2_*C*H_2_), 28.37 (CL*-CL, OCH_2_*C*H_2_), 25.64 (HL, OCOCH_2_*C*H_2_), 24.83 (CL, OCOCH_2_*C*H_2_), 24.59 (CL, OCOCH_2_CH_2_*C*H_2_); GPC (CHCl_3_, PMMA standard): *M*_n_ = 10.8 kg mol^−1^, *Đ*_M_ = 1.16.

### Synthesis of P(CL-b-HL) via ring-opening polymerization

In an inert nitrogen glovebox, catalyst Mg(BHT)_2_(THF)_2_ (8.0 mg, 13 μmol), PCL_32_ (100 mg, 27 μmol), HL (121 mg, 94.5 μmol) and dry toluene (1.0 ml) were charged into an ampoule. The ampoule was sealed for 1 h. The reaction was quenched by the addition of acidified (5% HCl) methanol. Chloroform was added to dissolve solids and the copolymer was precipitated in excess methanol three times, yielding the P(CL_32_-b-HL_33_) block core.

P(CL_32_-b-HL_33_): ^1^H NMR (400 MHz, CDCl_3_) *δ*/ppm: 4.06 (t, C*H*_*2*_O), 3.65 (2H, C*H*_*2*_OCO), 2.30 (t, OCOC*H*_*2*_), 1.73–1.33 (m, C*H*_*2*_C*H*_*2*_C*H*_*2*_ from CL; C*H*_*2*_C*H*_*2*_C*H*_*2*_C*H*_*2*_ from HL); 173.77 (HL*-HL, O*C*OCH_2_), 173.56 (CL*-CL, O*C*OCH_2_), 64.28 (HL*-HL, O*C*H_2_), 64.12 (CL*-CL, O*C*H_2_), 34.21 (HL*-HL, OCO*C*H_2_), 34.14 (CL*-CL, OCO*C*H_2_), 28.78 (HL*-HL, OCH_2_*C*H_2_), 28.37 (CL*-CL, OCH_2_*C*H_2_), 25.64 (HL, OCOCH_2_*C*H_2_), 24.83 (CL, OCOCH_2_*C*H_2_), 24.59 (CL, OCOCH_2_CH_2_*C*H_2_); GPC (CHCl_3_, PMMA standard): *M*_n_ = 12.4 kg mol^−1^, *Đ*_M_ = 1.18.

### Chain extension of crystalline core-forming blocks through RAFT polymerization

The synthetic route for the block copolymers used in this work is shown in Supplementary Scheme 1. Typically, PHL_50_ (100 mg, 0.0154 mmol), DMA (381 mg, 3.85 mmol) and AIBN (0.48 mg, 0.0031 mmol) were dissolved in 1,4-dioxane (2 ml) and placed in an ampoule. The solution was then freeze–pump–thawed three times and heated for 2 h at 70 °C. The reaction was quenched by immersion of the ampoule in liquid nitrogen, and the block polymer was precipitated in ice-cold diethyl ether three times before being dried under vacuum, yielding PHL_50_-b-PDMA_217_.

PHL_50_-b-PDMA_217_: ^1^H NMR (400 MHz, CDCl_3_) *δ*/ppm: 4.03 (t, C*H*_*2*_O (PHL)), 3.47–2.43 (m, N(C*H*_*3*_)_2_, C*H*CH_2_ from PDMA), 2.28–1.23 (m, OCOC*H*_*2*_ (PHL), C*H*_*2*_C*H*_*2*_C*H*_*2*_C*H*_*2*_ (PHL), CHC*H*_*2*_ (PDMA)); *M*_n_ = 28.6 kg mol^−1^, *Đ*_M_ = 1.24.

The chain extension RAFT polymerizations of other crystalline cores were completed by a similar method. Compositions were calculated from ^1^H NMR spectra, as shown in Supplementary Figs. 24 and 25.

### Synthesis of BODIPY-dye-functionalized homopolymers

Three different carboxylic-functionalized BODIPY dyes (BODIPY 630/650, BOPIPY R6G and BODIPY FL) were selected to modify three different core domains of PHL, PCL and P(VL-co-CL), respectively (Supplementary Scheme 2). Typically, with BODIPY 630/650 and PHL as an example, PHL_40_ (50 mg, 9.3 μmol), BODIPY 630/650 (3.3 mg, 9.3 μmol), 4-dimethylaminopyridine (0.56 mg, 4.6 μmol) and *N*-(3-dimethylaminopropyl)-*N*′-ethylcarbodiimide hydrochloride (17.9 mg, 93 μmol) were dissolved in 2 ml of dichloromethane and stirred for 24 h. The reaction mixture was precipitated in cold ethanol three times to yield the pure end-functionalized fluorescently labelled polymer, PHL_40_-R.

PHL_40_-R: ^1^H NMR (400 MHz, CDCl_3_) *δ*/ppm: 4.06 (t, C*H*_*2*_O (PHL)), 2.30 (t, OCOC*H*_*2*_ (PHL)), 1.73–1.33 (m, C*H*_*2*_C*H*_*2*_C*H*_*2*_C*H*_*2*_ (PHL)), 7.65–5.67 (m, 15H, aromatic (BODIPY)), 4.96 (s, 2H, OCOC*H*_*2*_O (BODIPY)).

Similarly, BOPIPY R6G-modified PCL was prepared (PCL-G), as well as BODIPY FL-modified P(VL-co-CL) (P(VL_30_-co-CL_35_)-B).

### Preparation of 1D short crystalline seed micelles

Typically, with PCL_62_-b-PDMA_270_ as an example, PCL_62_-b-PDMA_270_ (20 mg) was added to 4 ml of ethanol (5.0 mg ml^−1^) in a 7-ml vial. The samples were heated at 70 °C without stirring for 3 h, before cooling to room temperature (25 °C). The sample was then aged for five days at room temperature to yield micrometre-long polydisperse cylinders. Finally, the cylinders were sonicated using a Bandelin Sonopuls sonication probe in an ice bath for 20 min to yield short crystalline seeds.

### Living CDSA of homopolymer/BCP blends

Typically, with PHL_40_/PHL_50_-b-PDMA_217_ as an example, to five separate 7-ml screw cap vials was added 1 ml of a 0.01 mg ml^−1^ ethanol solution of PHL_50_-b-PDMA_217_ seed micelles. Varying amounts of PHL_40_/PHL_50_-b-PDMA_217_ blend unimer (1:1, wt/wt) dissolved in CHCl_3_ (10 mg ml^−1^) (50 μg, 100 μg, 150 μg, 200 μg and 250 μg, respectively) were then added. After shaking the vials for 10 s, the solutions were annealed at room temperature for one day before TEM imaging. Multiple TEM images were captured, and the contour areas were measured from ~100 micelles. Living CDSA of other cores was performed using the same method.

### Epitaxial growth

Typically, homopolymer and corresponding block copolymer (1:1, wt/wt) dissolved in CHCl_3_ (10 mg ml^−1^) was added to an ethanol dispersion of 1D seed micelles (0.01 mg ml^−1^) or 2D platelet micelles (0.02 mg ml^−1^) and aged for one day at ambient temperature (unless otherwise specified) before TEM and AFM analysis.

### Statistics of the area of 2D platelets or length of 1D seeds

The number-average area (*A*_n_) and weight-average area (*A*_w_) of platelets were calculated from more than 100 platelets using the software ImageJ. Values of *A*_n_ and *A*_w_ were calculated using the equations

$${A_n} = {\frac{{\mathop {\sum}\nolimits_{i = 1}^{i = n} {A_in_i} }}{{\mathop {\sum}\nolimits_{i = 1}^{i = n} {n_i} }}}$$

$${A_w} = {\frac{{\mathop {\sum}\nolimits_{i = 1}^{i = n} {A_i^2n_i} }}{{\mathop {\sum}\nolimits_{i = 1}^{i = n} {A_in_i} }}}$$

where *n*_*i*_ is the number of platelets, *A*_*i*_ is the area of the platelets, and *n* is the total number of platelets examined for each sample. The distribution of platelet area is characterized by *A*_*w*_*/A*_*n*_. The statistics for the length analysis of the 1D seed micelles was achieved using the same method.

### Selective degradation of the multiblock co-micelles

The as-prepared multiblock co-micelles in ethanol were first dialysed against water, then KOH was added to achieve a final concentration of KOH of 1 M. At certain time intervals, 1 ml of multiblock co-micelle solution was removed and dialysed against water to completely remove the KOH. The dialysate was then analysed by TEM and AFM imaging.

## Online content

Any methods, additional references, Nature Portfolio reporting summaries, source data, extended data, supplementary information, acknowledgements, peer review information; details of author contributions and competing interests; and statements of data and code availability are available at 10.1038/s41557-023-01177-2.

## Supplementary information

Supplementary Information Supplementary Schemes 1 and 2, Tables 1 and 2, Figs. 1–26 and text.

## Data Availability

The data supporting the findings of this study are available within this paper and its Supplementary Information. Source data are available from 10.6084/m9.figshare.21603813.
