# Supplementary material for: Uniform segmented platelet micelles with compositionally distinct and selectively degradable cores
Source: Nat Chem. 2023 Apr 20;15(6):824–31. doi: 10.1038/s41557-023-01177-2 (PMC10239731; doi:10.1038/s41557-023-01177-2)
Supplement: Supplementary file 1 — Supplementary Schemes 1 and 2, Tables 1 and 2, Figs. 1–26 and text. [file 41557_2023_1177_MOESM1_ESM.pdf]

nature chemistry

Article
https://doi.org/10.1038/s41557-023-01177-2

Uniform segmented platelet micelles with compositionally distinct and selectively degradable cores

In the format provided by the
authors and unedited

Table of Contents:

Supplementary Schemes 1 and 2

Supplementary Tables 1 and 2

Supplementary Figures 1 to 26

Supplementary Text

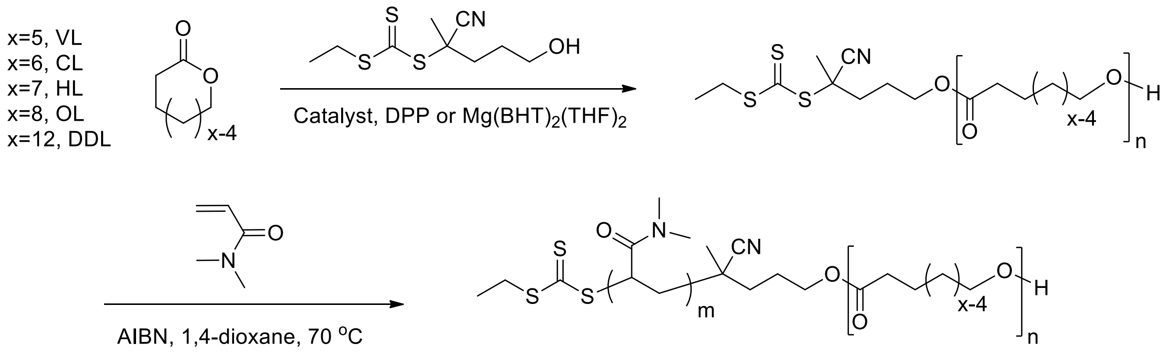

Supplementary Scheme 1. The synthetic route of block copolymers used in this work.

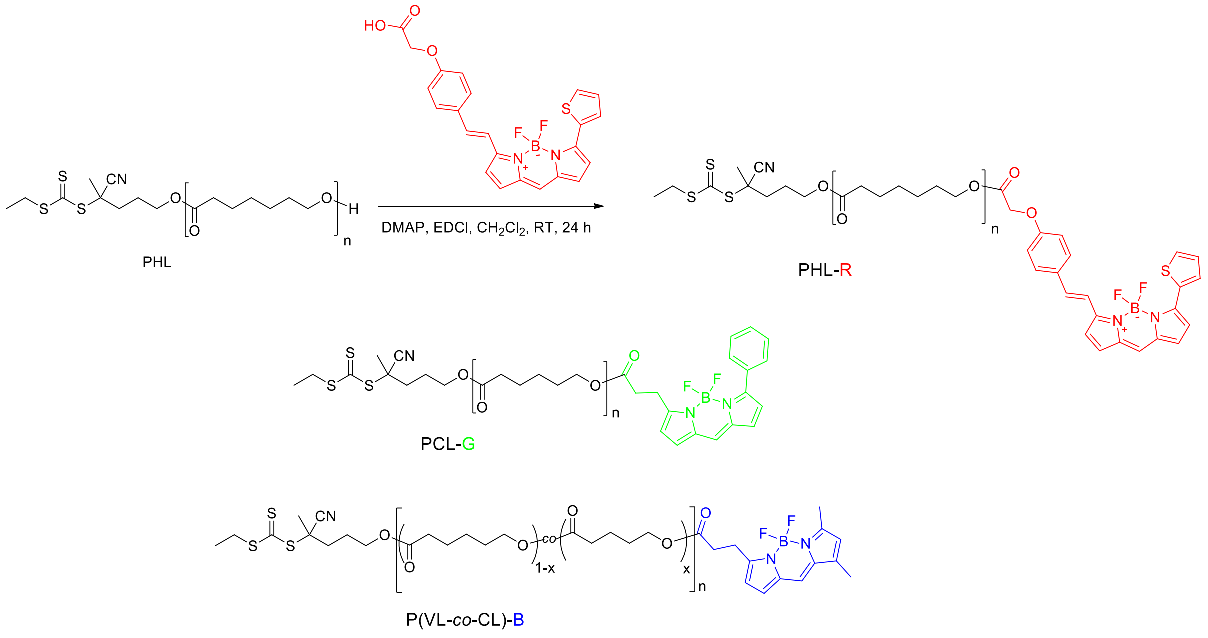

Supplementary Scheme 2. The synthetic routine of PHL-R (Red) with BODIPY 630/650 dye and the structure of PCL-G (Green) and P(VL-*co*-CL)-B (Blue) are also shown.

Supplementary Table 1. Molecular characteristics of the polymers used in this study.

**Polymer (NMR)** [a]	$M_{n, NMR}$ [a] **(kDa)**	$M_{n, GPC}$ [b] **(kDa)**	$Đ_M$ [b]
**PCL_{40}**	4.8	7.6	1.09
**PCL_{62}**	7.3	12.3	1.10
**PCL_{62}-*b*-$PDMA_{270}$**	34.0	32.5	1.21
**PVL_{80}**	8.2	9.8	1.22
**PVL_{80}-*b*-$PDMA_{295}$**	37.4	34.5	1.20
**P(VL_{30}-*co*-CL_{35})**	7.2	11.8	1.17
**P(VL_{30}-*co*-CL_{35})-*b*-$PDMA_{80}$**	15.2	16.6	1.19
**P(VL_{30}-*co*-CL_{35})-*b*-$PDMA_{265}$**	33.5	30.7	1.24
**PHL_{40}**	5.4	7.4	1.16
**PHL_{50}**	6.5	8.3	1.17
**PHL_{50}-*b*-$PDMA_{217}$**	28.1	28.6	1.24
**P(CL_{32}-*co*-HL_{28})**	7.5	10.8	1.16
**P(CL_{32}-*co*-HL_{28})-*b*-$PDMA_{67}$**	17.4	17.0	1.18
**P(CL_{32}-*co*-HL_{28})-*b*-$PDMA_{268}$**	34.0	33.4	1.22
**P(CL_{32}-*b*-HL_{33})**	8.1	12.4	1.18
**P(CL_{32}-*b*-HL_{33})-*b*-$PDMA_{178}$**	30.0	31.0	1.23
**POL_{55}**	7.8	11.5	1.34
**POL_{55}-*b*-$PDMA_{280}$**	35.6	29.0	1.18
**$PDDL_{40}$**	10.9	12.8	2.21
**$PDDL_{40}$-*b*-$PDMA_{260}$**	37.2	35.8	1.35

a) The polymer compositions were determined from ^{1}H NMR spectra;
b) The polydispersity was obtained from GPC analysis with $CHCl_3$ as the eluent.

Supplementary Table 2. *d*-spacings of different polylactone homopolymers calculated from WAXD profiles.

**Polymer** [a]	**PVL_{80} (C_5)**		**PCL_{40} (C_6)**		**PHL_{50} (C_7)**		**POL_{55} (C_8)**		**$PDDL_{40}$ (C_{12})**	
**Crystalline plane**	(110)	(200)	(110)	(200)	(110)	(200)	(110)	(200)	(110)	(200)
**2θ**	21.7	24.4	21.6	24.0	21.6	24.3	21.6	24.1	21.6	24.0
***d*-spacing (nm)**	0.409	0.364	0.412	0.371	0.412	0.365	0.413	0.369	0.413	0.371

a) Powder samples were used for WAXD measurement.

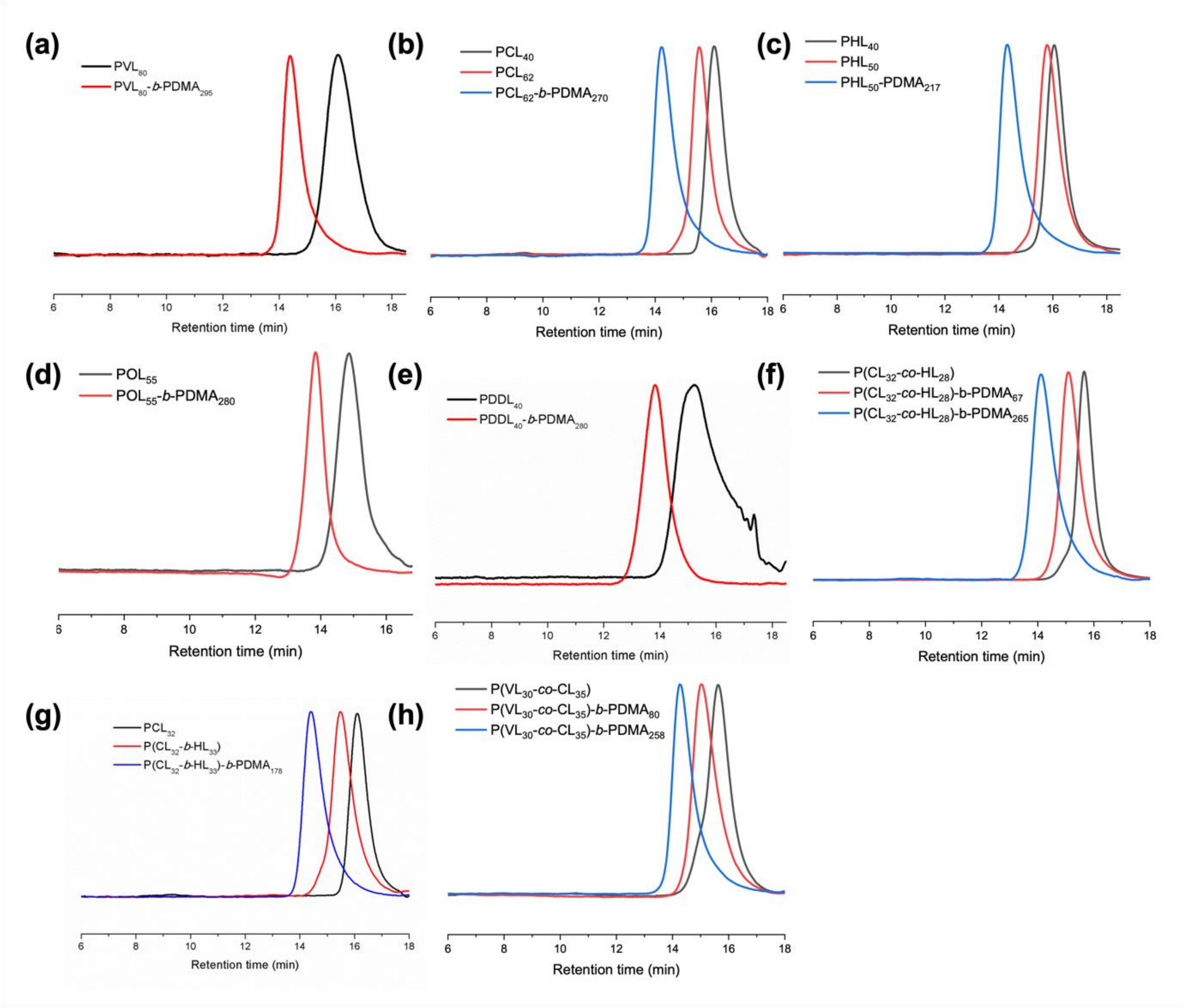

Supplementary Fig. 1. Overlaid gel permeation chromatograms (refractive index detector, $CHCl_3$ with 0.5% trimethylamine (TEA) as an eluent, 40 °C PMMA standards) for different polymers used in this study. (a) PVL, **(b)** PCL, **(c)** PHL, **(d)** POL, **(e)** PDDL, **(f)** P(CL-*co*-HL), **(g)** P(CL-*b*-HL) and **(h)** P(VL-*co*-CL)-based homopolymers and block copolymers.

The peak of PDDL GPC curve (**Supplementary Fig. 1e**) at about 17 min is attributed to the signal of PDDL homopolymer with small molecular weight. Since DDL is a much larger (13-membered) cyclic molecule compared to CL (7-membered cyclic molecule) with a lower ring-strain that results from its greater flexibility, the ring-opening polymerization of DDL is driven by the entropic gain of rotation from ring-opening rather than ring strain enthalpy, thus resulting in a relatively high molecular weight distribution. Similar result is also observed in reported papers for ring opening polymerization with a larger ring monomer (*43*,*45*).

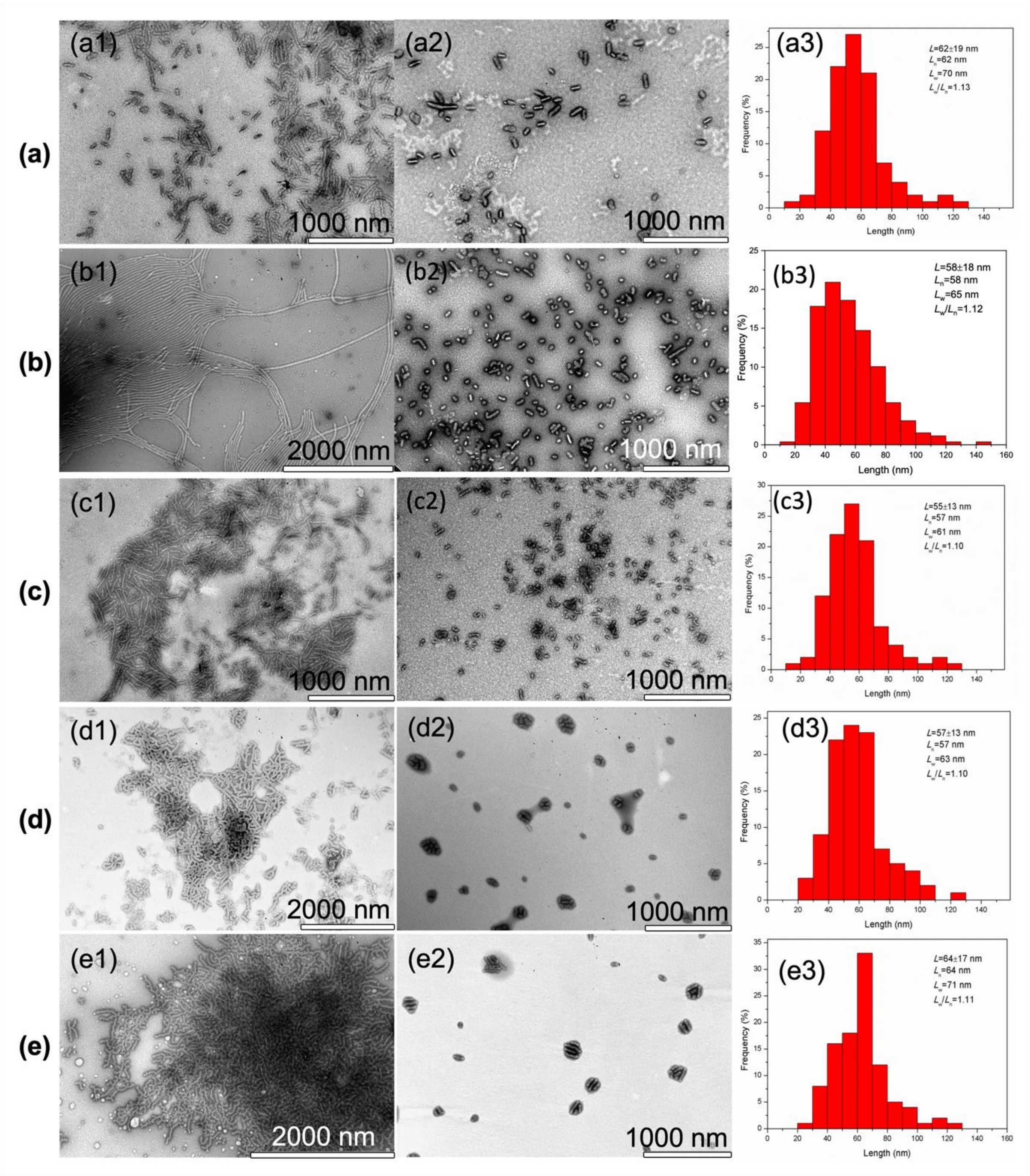

Supplementary Fig. 2. Preparation of 1D seeds of different core-forming BCPs. Left: Polydisperse cylinders of different core-forming BCPs prepared from spontaneous nucleation in ethanol at 5 mg/mL, i.e. heating at 70 °C for 3 h then subsequently cooling to room temperature (25 °C) before aging for 5 days; middle: corresponding uniform 1D crystalline seeds after sonication for 20 min; right: corresponding length statistics of the 1D short seeds. **(a)** PVL_{80}-*b*-$PDMA_{295}$, **(b)** PCL_{62}-*b*-$PDMA_{270}$, **(c)** PHL_{50}-*b*-$PDMA_{217}$, **(d)** POL_{55}-*b*-$PDMA_{280}$ and **(e)** $PDDL_{40}$-*b*-$PDMA_{260}$.

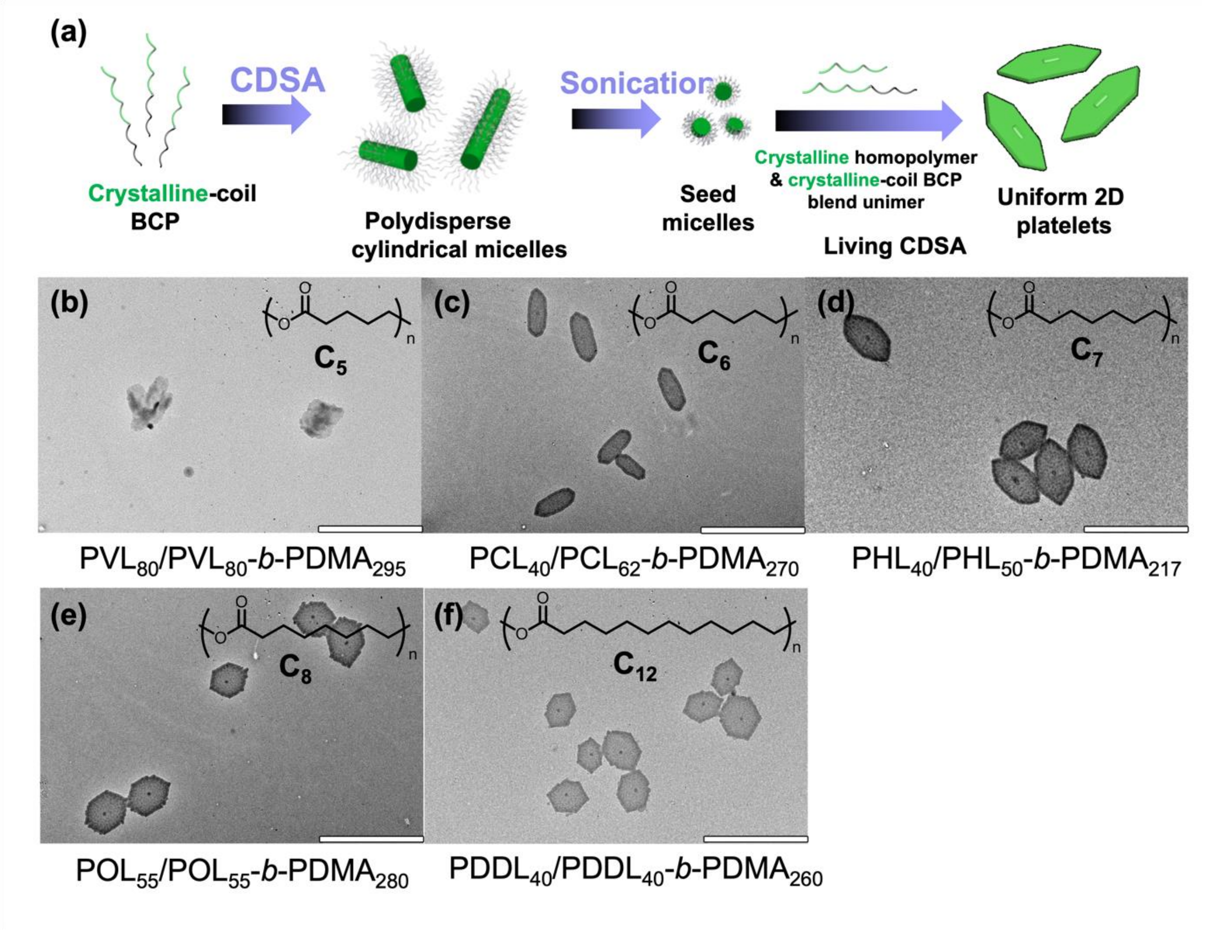

Supplementary Fig. 3. Assemblies obtained by homoepitaxial growth of different poly(lactones) homopolymer and block copolymer blends (1:1, w/w) from corresponding 1D seeds with the same crystalline core. (a) Schematic illustration of this process. TEM images of 2D structures of **(b)** PVL (C_5), **(c)** PCL (C_6), **(d)** PHL (C_7), **(e)** POL (C_8) and **(f)** PDDL (C_{12}). The original seeds can be clearly visualized in the centre of well-developed platelets for PCL, PHL, POL and PDDL. Insets show the chemical structures of different cores. Scale bars = 2000 nm.

The formation of poor-developed 2D PVL platelets is probably attributed to the relatively faster micellization rate compared to the crystallization rate of PVL/PVL-*b*-PDMA. It is noted that the final morphology of crystalline BCPs is a result of the competition between crystallization and micellization. The PVL core exhibits the lowest crystallization temperature compared with other polylactone cores (**Supplementary Fig. 26**), indicating the relatively low crystallization ability of PVL (low supercooling degree for crystallization), and on the other hand, a relatively fast micellization rate. Although many attempts are tried to alleviate the micellization ability of PVL/PVL-*b*-PDMA blend unimer such as changing the polymerization degree of PVL/PDMA blocks, selective solvents and crystallization temperature, it seems that decreasing the crystallization temperature (0 °C, increasing the crystallization rate) is somewhat beneficial for the epitaxial growth of PVL (**Supplementary Fig. 12**). However, the morphologies of these platelets are still not as regular as the other core-forming platelets and some spherical micelles are also observed for the seeded growth of PVL/PVL-*b*-PDMA (**Supplementary Fig. 12**), which is evidence for the fast micellization of PVL/PVL-*b*-PDMA blend unimer.

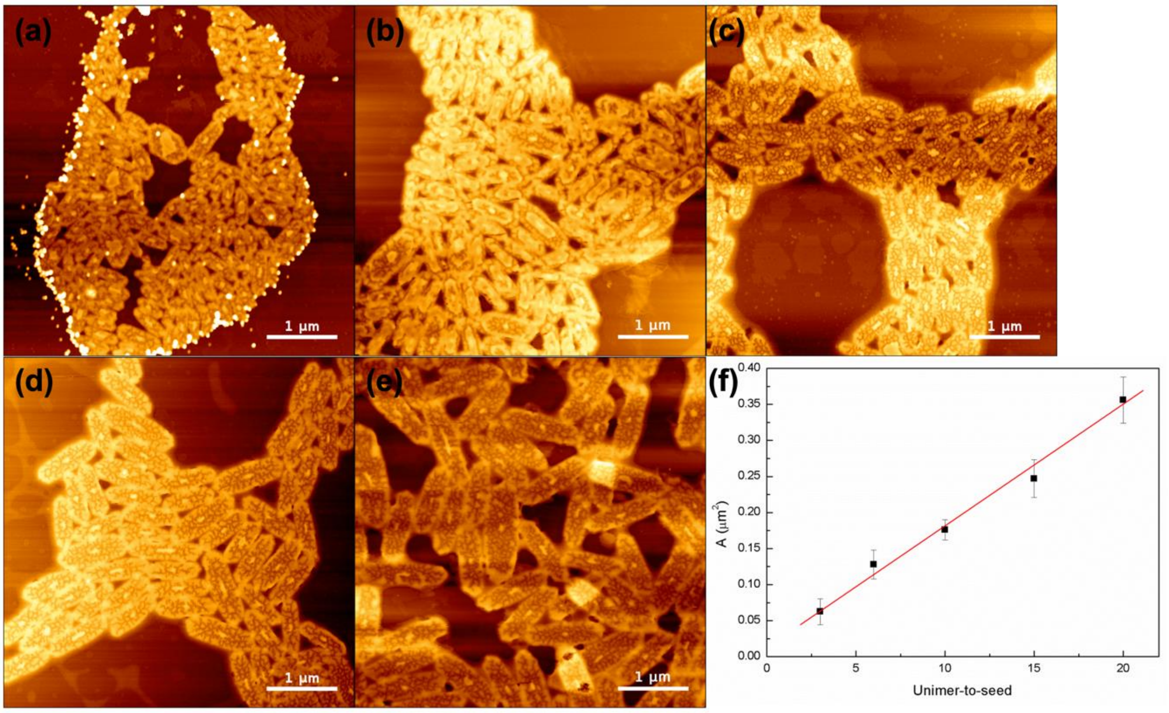

Supplementary Fig. 4. Living CDSA of PCL_{40}/PCL_{62}-*b*-$PDMA_{270}$ (1:1, w/w) blend from 1D PCL_{62}-*b*-$PDMA_{270}$ seeds. (a-e) AFM height images of 2D platelets of PCL_{40}/PCL_{62}-*b*-$PDMA_{270}$ (1:1, w/w, 10 mg/mL in $CHCl_3$) grown from 1D crystalline seeds of PCL_{62}-*b*-$PDMA_{270}$ (0.01 mg/mL, 1 mL) with m_{unimer} to m_{seed} (unimer-to-seed) ratios of **(a)** 3, **(b)** 6, **(c)** 10, **(d)** 15 and **(e)** 20. **(f)** Plots of area of 2D platelet against unimer-to-seed ratios. Data are presented as mean values +/- standard deviation (n = 100).

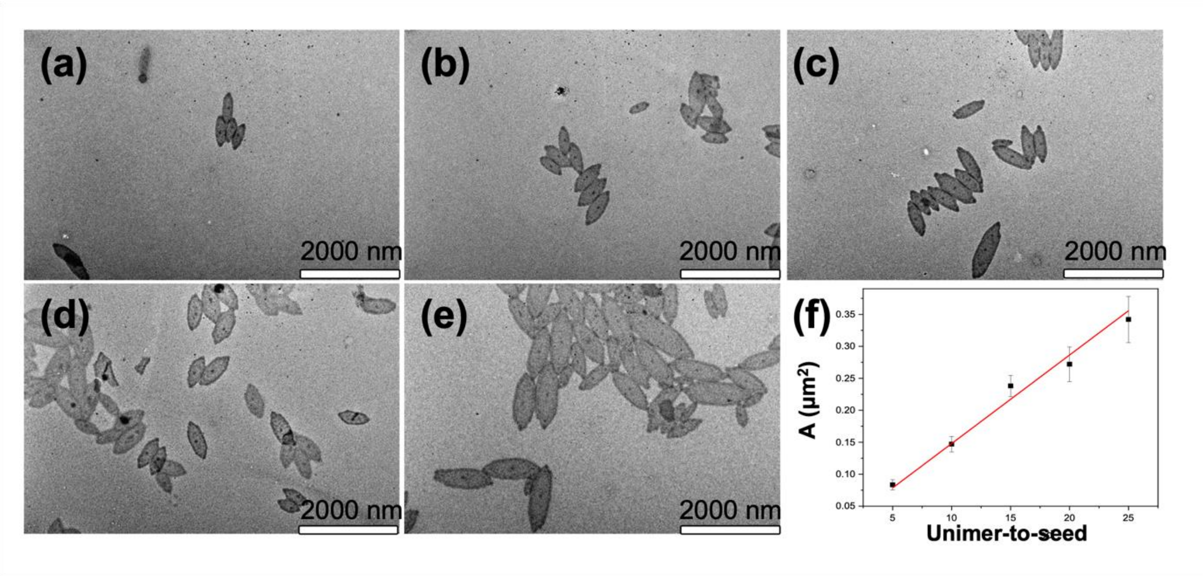

Suppplementary Fig. 5. Living CDSA of PHL_{40}/PHL_{50}-*b*-$PDMA_{217}$ (1:1, w/w) blend from 1D PHL_{50}-*b*-$PDMA_{217}$ seeds. TEM micrographs of 2D platelets of PHL_{40}/PHL_{50}-*b*-$PDMA_{217}$ blend (1:1, w/w, 10 mg/mL in $CHCl_3$) grown from 1D crystalline seeds of PHL_{50}-*b*-$PDMA_{217}$ (0.01 mg/mL, 1 mL) with unimer-to-seed ratios of **(a)** 5, **(b)** 10, **(c)** 15, **(d)** 20 and **(e)** 25. **(f)** Plot of 2D platelet area against unimer-to-seed ratios. Data are presented as mean values +/- standard deviation (n = 100).

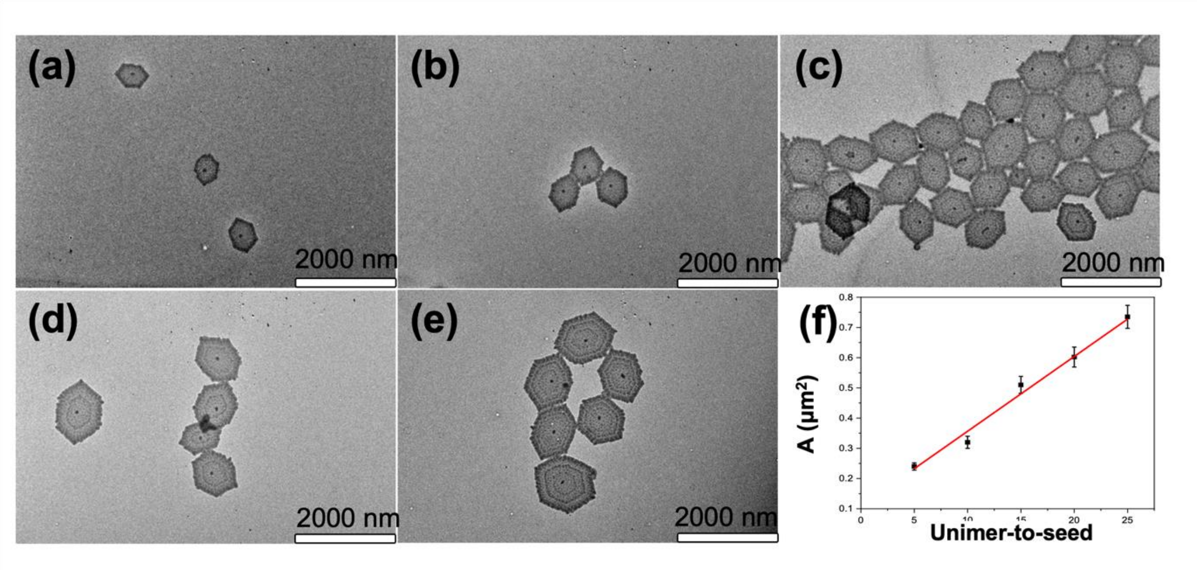

Supplementary Fig. 6. Living CDSA of POL_{55}/POL_{55}-*b*-$PDMA_{280}$ (1:1, w/w) blend from 1D POL_{55}-*b*-$PDMA_{280}$ seeds. TEM micrographs of 2D platelets of POL_{55}/POL_{55}-*b*-$PDMA_{280}$ blend (1:1, w/w, 10 mg/mL in $CHCl_3$) grown from 1D POL_{55}-*b*-$PDMA_{280}$ crystalline seeds (0.01 mg/mL, 1 mL) with unimer-to-seed ratios of **(a)** 5, **(b)** 10, **(c)** 15, **(d)** 20 and **(e)** 25. **(f)** Plot of 2D platelet area against unimer-to-seed ratios. Data are presented as mean values +/- standard deviation (n = 100).

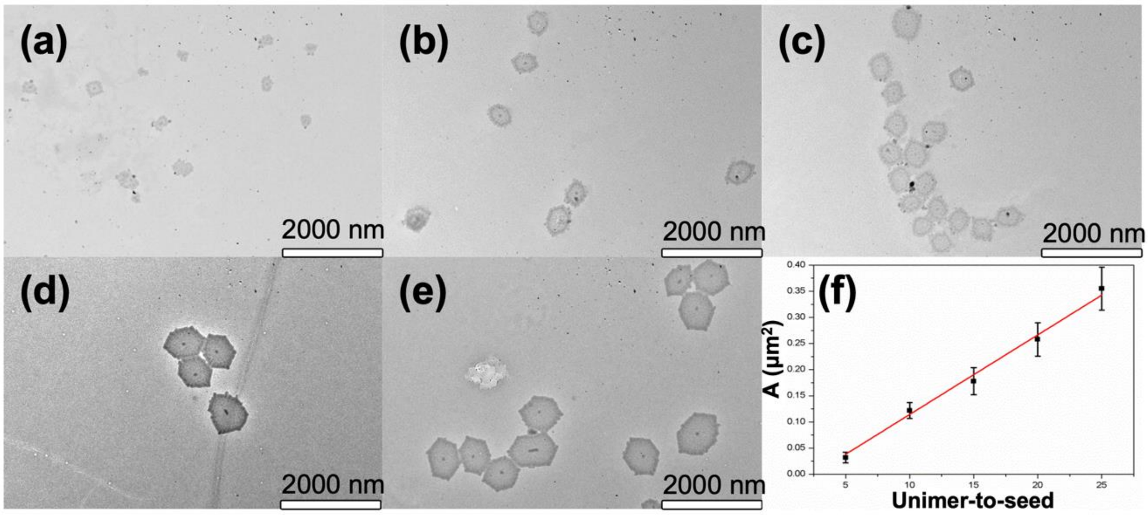

Supplementary Fig. 7. Living CDSA of $PDDL_{40}$/$PDDL_{40}$-*b*-$PDMA_{260}$ (1:1, w/w) blend from 1D $PDDL_{40}$-*b*-$PDMA_{260}$ seeds. TEM micrographs of 2D platelets of $PDDL_{40}$/$PDDL_{40}$-*b*-$PDMA_{260}$ blend (1:1, w/w, 10 mg/mL in $CHCl_3$) grown from 1D $PDDL_{40}$-*b*-$PDMA_{260}$ crystalline seeds (0.01 mg/mL, 1 mL) with unimer-to-seed ratios of **(a)** 5, **(b)** 10, **(c)** 15, **(d)** 20 and **(e)** 25. **(f)** Plot of 2D platelet area against unimer-to-seed ratios. Data are presented as mean values +/- standard deviation (n = 100).

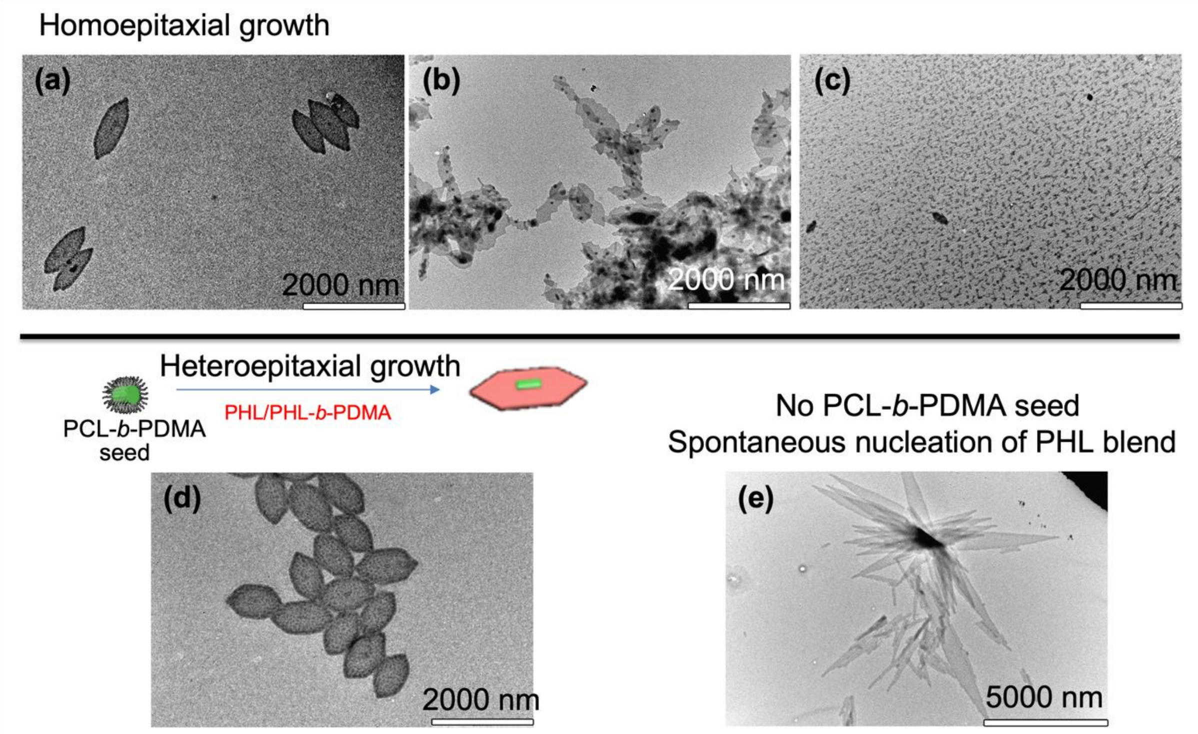

Supplementary Fig. 8. Control experiments and epitaxy. TEM morphologies of **(a)** addition of PHL_{40}/PHL_{50}-*b*-$PDMA_{217}$ blend unimer (1:1, w/w) in $CHCl_3$ (0.1 mg, 10 mg/mL) to the 1D PHL_{50}-*b*-$PDMA_{217}$ seeds (0.01 mg/mL, 1 mL); **(b)** addition of only PHL_{40} unimer in $CHCl_3$ (0.1 mg, 10 mg/mL) to the 1D PHL_{50}-*b*-$PDMA_{2170}$ seeds (0.01 mg, 1 mL); **(c)** addition of only PHL_{50}-*b*-$PDMA_{217}$ unimer in $CHCl_3$ (0.1 mg, 10 mg/mL) to the 1D PHL_{50}-*b*-$PDMA_{217}$ seeds (0.01 mg, 1mL); **(d)** addition of PHL_{40}/PHL_{50}-*b*-$PDMA_{217}$ blend unimer (1:1, w/w) in $CHCl_3$ (0.1 mg, 10 mg/mL) to the 1D PCL_{62}-*b*-$PDMA_{270}$ seeds (0.01 mg, 1 mL) for initiating growth; **(e)** addition of PHL_{40}/PHL_{50}-*b*-$PDMA_{217}$ blend unimer (1:1, w/w) in $CHCl_3$ (0.1 mg, 10 mg/mL) to ethanol solvent without any seeds (by spontaneous nucleation).

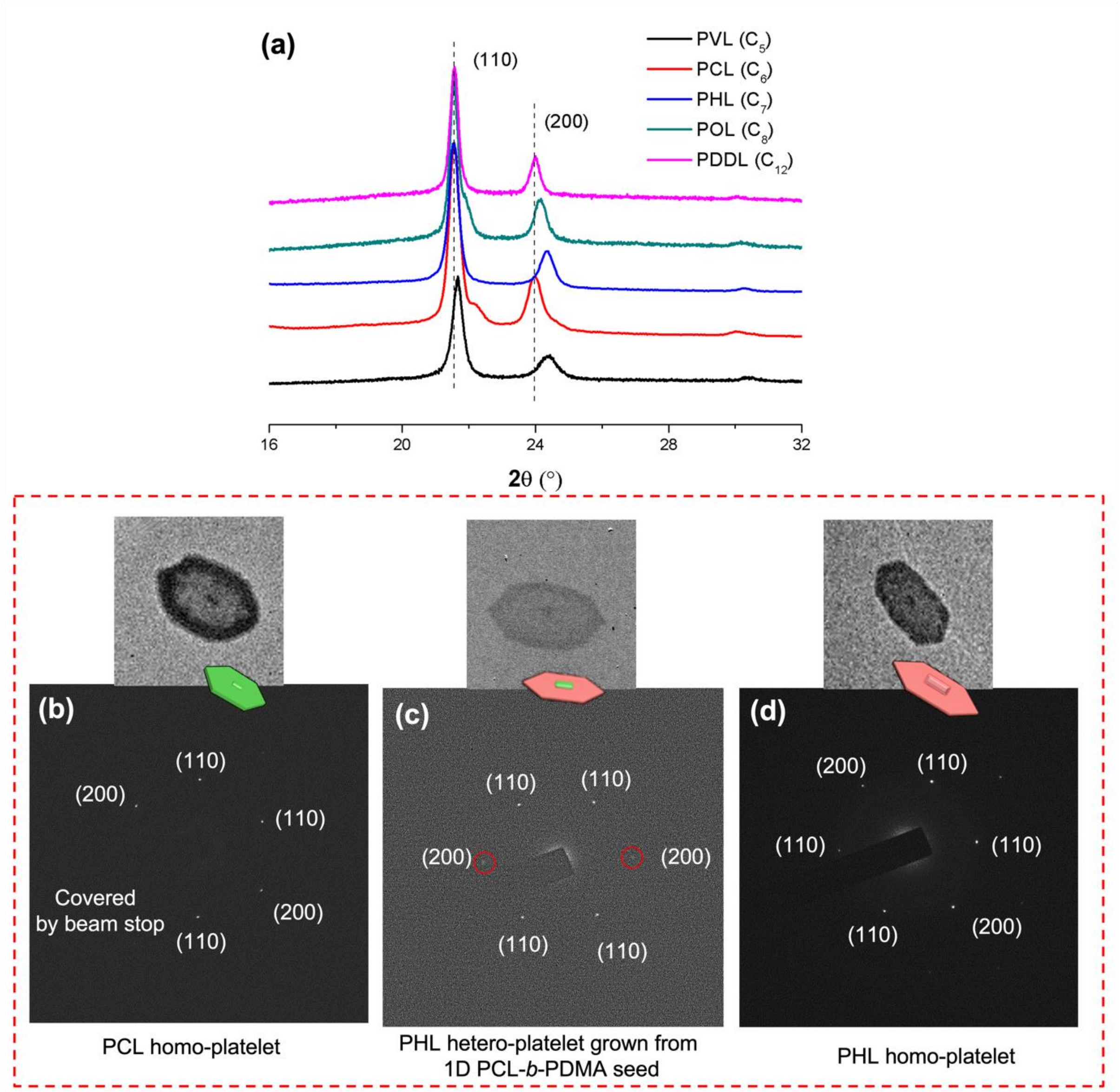

Supplementary Fig. 9. Analysis of crystalline structure of platelet micelles. (a) WAXD profiles of powder samples for different homopolymers. TEM and corresponding SAED images of **(b)** 2D PCL homo-platelet grown from a PCL_{62}-*b*-$PDMA_{270}$ seed (noting that one (110) diffraction spot is covered by beam stop), **(c)** 2D PHL segmented platelet micelles grown from a PCL_{62}-*b*-$PDMA_{270}$ seed and **(d)** 2D PHL homo-platelet homoepitaxially grown from a PHL_{50}-*b*-$PDMA_{217}$ seed.

WAXD result shows *d*-spacing of (110) and (200) of PCL powder sample is 0.412 nm and 0.371 nm, respectively, and 0.412 nm and 0.365 nm for PHL powder sample, respectively (**Supplementary Table 2**).

Analysis of SAED pattern of **(b)** shows that the *d*-spacing of (110) and (200) of PCL platelet is 0.412 nm and 0.371 nm, respectively, while SAED patterns of **(c)** and **(d)** show the *d*-spacing of (110) and (200) of both PHL platelets grown from either a PCL_{62}-*b*-$PDMA_{270}$ seed or a PHL_{50}-*b*-$PDMA_{217}$ seed is 0.412 nm and 0.365 nm, respectively. The associated *d*-spacing of (200) of PHL platelets grown from 1D PCL_{62}-*b*-$PDMA_{270}$ seeds has an identical value as that of PHL platelets homoepitaxially grown from 1D PHL_{50}-*b*-$PDMA_{217}$ seed, indicating that the crystalline structure of PHL platelets retain its inherent packing structure grown from 1D PCL_{62}-*b*-$PDMA_{270}$ seeds.

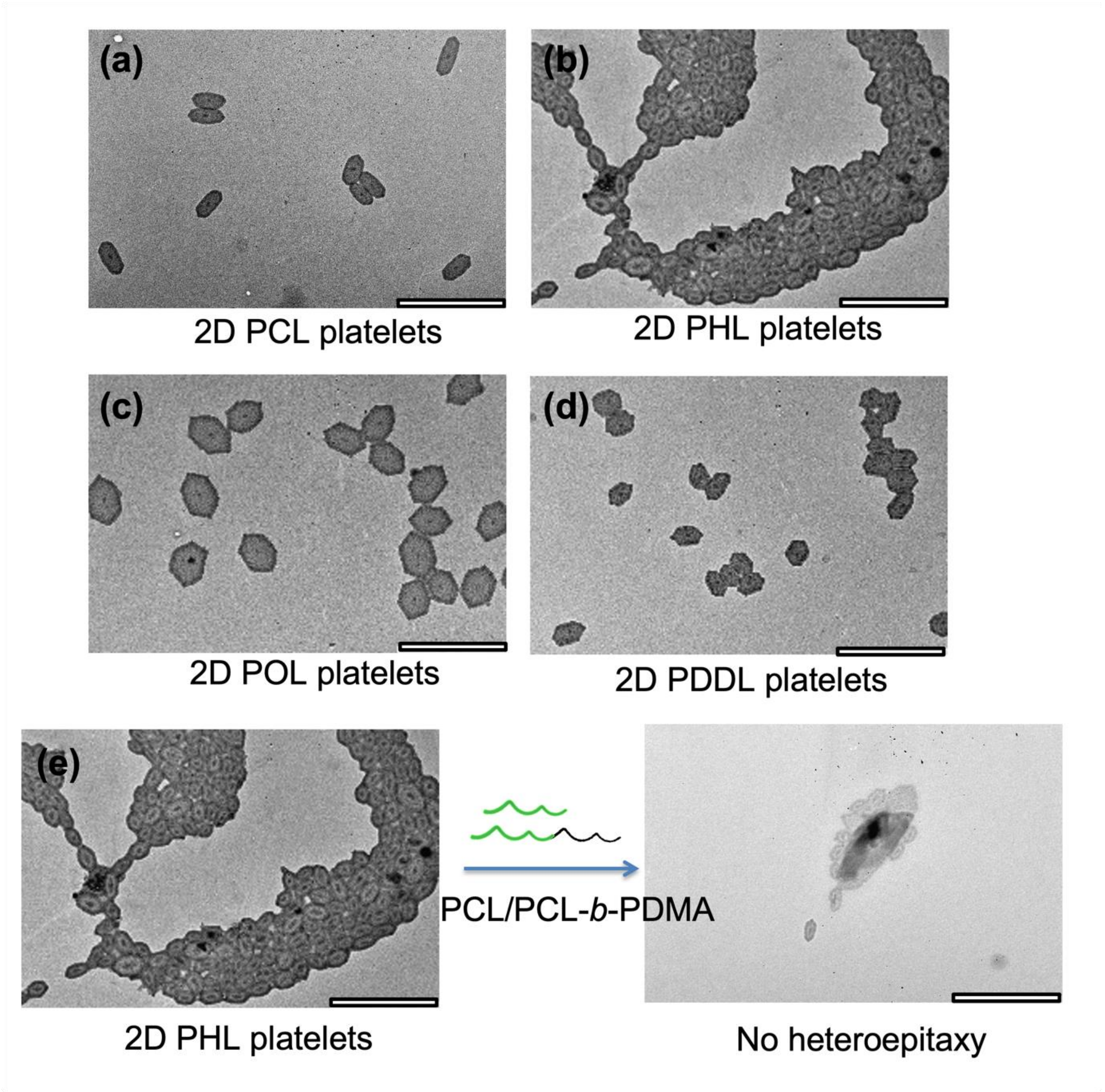

Supplementary Fig. 10. Four different core-forming 2D platelets as seeds to initial the heteroepitaxial growth for other core-forming blend unimers. TEM images of **(a)** PCL, **(b)** PHL, **(c)** POL and **(d)** PDDL platelets. **(e)** TEM morphology of addition of PCL core-forming blend unimer into the 2D PHL platelets and no heteroepitaxy was observed in this case. The weight ratio for homopolymer and block copolymer is fixed 1:1 in all cases. Scale bar = 2000 nm.

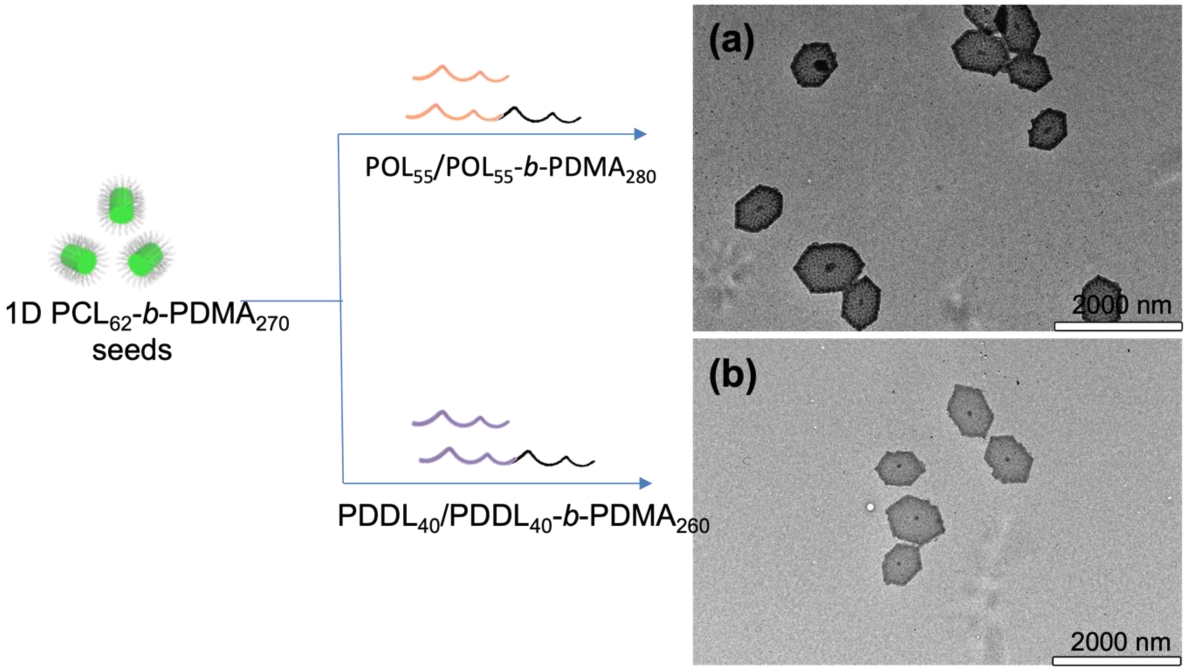

Supplementary Fig. 11. Growth of POL_{55}/POL_{55}-*b*-$PDMA_{280}$ and $PDDL_{40}$/$PDDL_{40}$-*b*-$PDMA_{260}$ unimer blends in the presence of 1D PCL_{62}-*b*-$PDMA_{270}$ seed micelles. TEM morphologies of adding **(a)** POL_{55}/POL_{55}-*b*-$PDMA_{280}$ and **(b)** $PDDL_{40}$/$PDDL_{40}$-*b*-$PDMA_{260}$ blend unimer (1:1, w/w, 0.1 mg, 10 mg/mL) in the presence of 1D PCL_{62}-*b*-$PDMA_{270}$ seeds (0.01 mg, 1 mL), respectively.

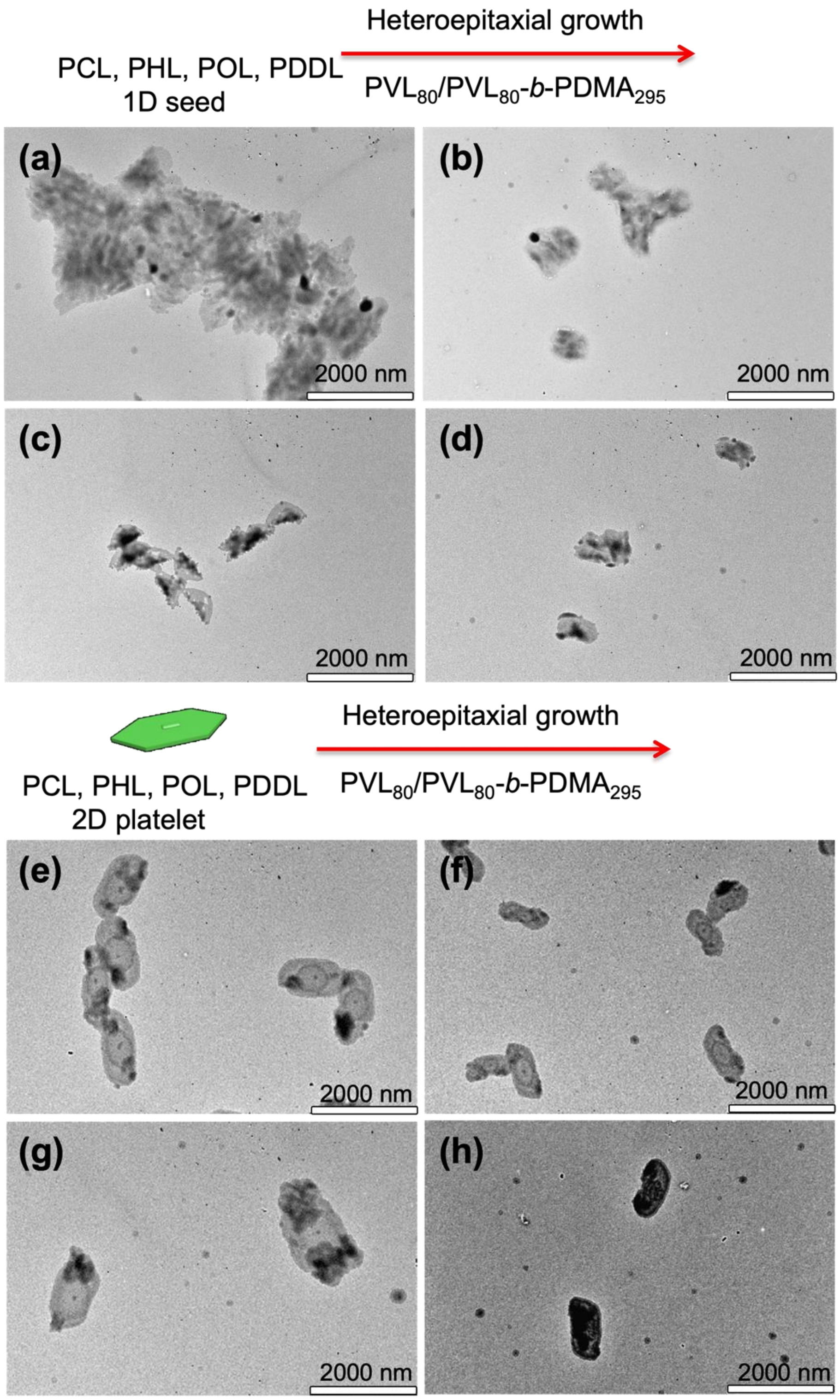

Supplementary Fig. 12. Growth of PVL_{80}/PVL_{80}-*b*-$PDMA_{295}$ blend unimer (1:1, w/w) in the presence of either 1D seeds or 2D platelets of the other cores. TEM morphologies of addition of PVL_{80}/PVL_{80}-*b*-$PDMA_{295}$ blend unimer (1:1, w/w in $CHCl_3$, 0.1 mg, 10 mg/mL) to 1D seed micelles of **(a)** PCL, **(b)** PHL, **(c)** POL, **(d)** PDDL (0.01 mg, 1 mL); and a solution containing 2D platelet micelles of **(e)** PCL, **(f)** PHL, **(g)** POL and **(h)** PDDL (0.033 mg, 1 mL). Note the crystallization temperature for PVL blend unimer was 0 °C.

For higher crystallization temperatures such as between 25 and 30 °C, no epitaxial growth of PVL was observed in all cases. In contrast, for lower crystallization temperatures between 0 and -25 °C, epitaxial growth of PVL blend unimer was observed but the formed 2D platelets were not well-developed as shown in **Supplementary Fig. 12**. The poor-defined PVL morphologies (**Supplementary Fig. 12a-d**) grown by seeded growth using 1D seeds of other cores are very similar to that grown from homoepitaxy of PVL (**Supplementary Fig. 3b**), confirming that the PVL system is unable to form well-developed 2D platelets under these assembly conditions. The reason could be probably attributed to the relatively faster micellization rate compared to crystallization rate of PVL/PVL-*b*-PDMA as mentioned above (**Supplementary Fig. 3b**). Although low crystallization temperature is somewhat beneficial for the epitaxial growth of PVL, the formed PVL platelets are still not as regular as the other core-forming platelets. Moreover, some spherical micelles are also observed for the seeded growth of PVL/PVL-*b*-PDMA (**Supplementary Fig. 12**), which is evidence for the fast micellization of PVL/PVL-*b*-PDMA blend unimer.

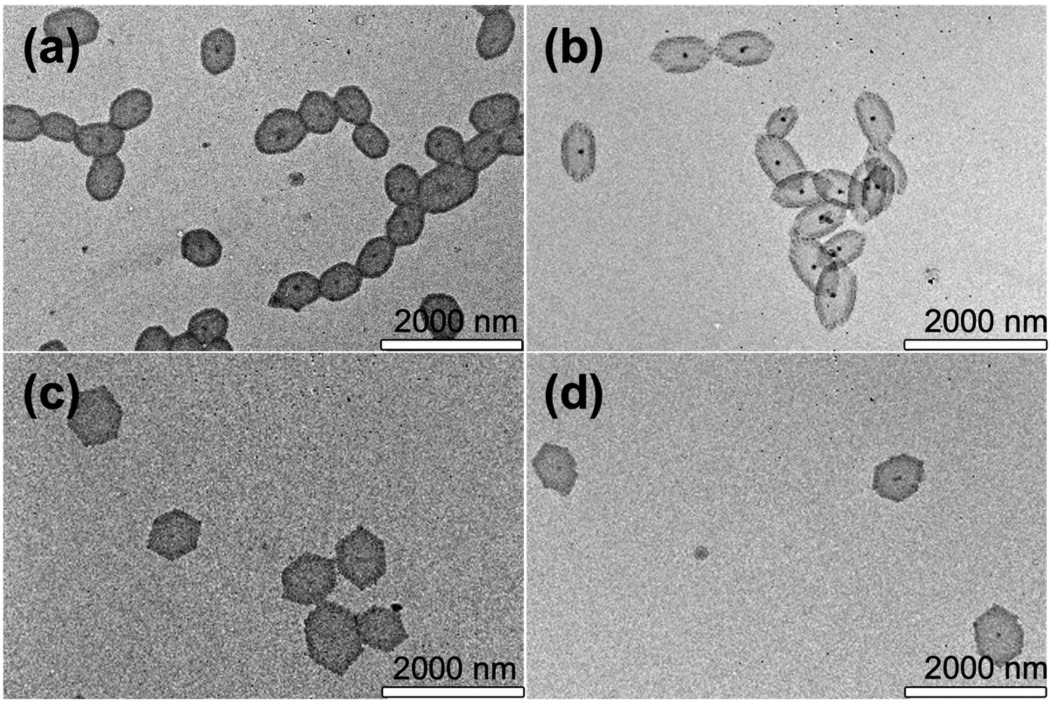

Supplementary Fig. 13. Growth of the complementary core blend unimer in the presence of 1D PVL_{80}-*b*-$PDMA_{295}$ seeds. TEM morphologies of 2D platelets prepared by adding **(a)** PCL_{40}/PCL_{62}-*b*-$PDMA_{270}$, **(b)** PHL_{40}/PHL_{50}-*b*-$PDMA_{217}$, **(c)** POL_{55}/POL_{55}-*b*-$PDMA_{280}$ and **(d)** $PDDL_{40}$/$PDDL_{40}$-*b*-$PDMA_{260}$ blend unimer (1:1, w/w, 0.1 mg, 10 mg/mL) into 1D PVL_{80}-*b*-$PDMA_{295}$ seeds (0.01 mg, 1 mL), respectively.

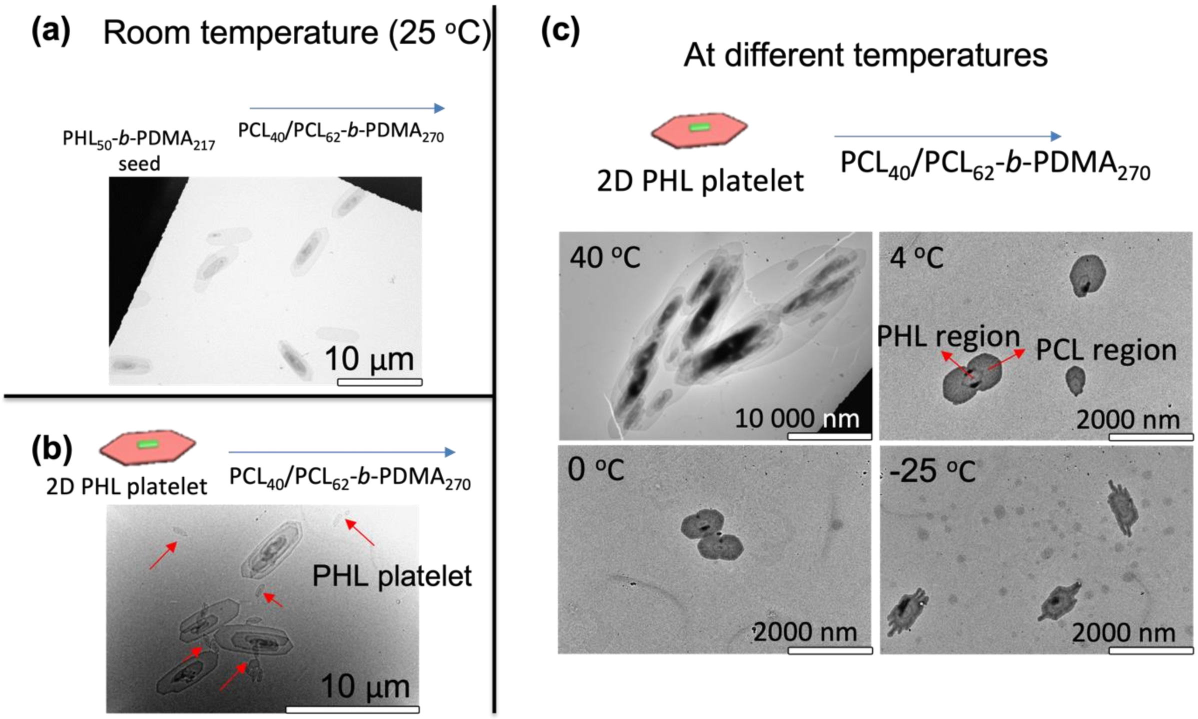

Supplementary Fig. 14. Effect of crystallization temperatures on the growth of PCL_{40}/PCL_{62}-*b*-$PDMA_{270}$ blend unimer (1:1, w/w) in the presence of 1D PHL_{50}-*b*-$PDMA_{217}$ seeds or 2D PHL platelets. (a) Addition of PCL_{40}/PCL_{62}-*b*-$PDMA_{270}$ blend unimer (1:1, w/w) in $CHCl_3$ (0.1 mg, 10 mg/mL) to the 1D PHL_{50}-*b*-$PDMA_{217}$ seeds (0.01 mg, 1 mL) at room temperature (25 °C); **(b)** addition of PCL_{40}/PCL_{62}-*b*-$PDMA_{270}$ blend unimer (1:1, w/w) in $CHCl_3$ (0.06 mg, 10 mg/mL) to the 2D PHL-based platelets (0.02 mg, 1 mL) at room temperature (25 °C); **(c)** addition of PCL_{40}/PCL_{62}-*b*-$PDMA_{270}$ blend unimer (1:1, w/w) in $CHCl_3$ (0.06 mg, 10 mg/mL) to 2D PHL-based platelets (0.02 mg, 1 mL) at different temperatures indicated. The results show that lower crystallization temperatures are beneficial for growth.

The temperature-dependent growth of PCL blend unimer from a PHL seed can be explained on the basis of Hoffmann−Lauritzen theory and a template model proposed by Greso and Phillips (*40*). It is well-documented that polymer crystals grow from nuclei and there exists a critical size (core thickness) for this to occur. Polymer crystallization can occur only when the nucleus reaches the critical size and epitaxial crystallization is also no exception. The critical nucleus size depends on the temperature at which polymer crystallization takes place. The higher the temperature the larger the critical nucleus size. The temperature-dependent growth of PCL blend unimer from a PHL seed can be explained in terms of discrepancies in their core thicknesses. The epitaxial crystallization of PCL with a larger core thickness than the PHL core thickness of the seeds cannot take place as it will not be energetically favourable. Considering that the preferred core thickness of PCL depends on its crystallization temperature, the value can be reduced by lowering the temperature. Consequently, spontaneous nucleation of PCL is observed at high temperature but epitaxy takes place at low temperature. Lattice matching is a necessary but insufficient criterion for epitaxial growth. A larger size for the seed crystals compared to the newly grown polymer crystals in the lattice matching direction is crucial for epitaxial crystallization to occur.

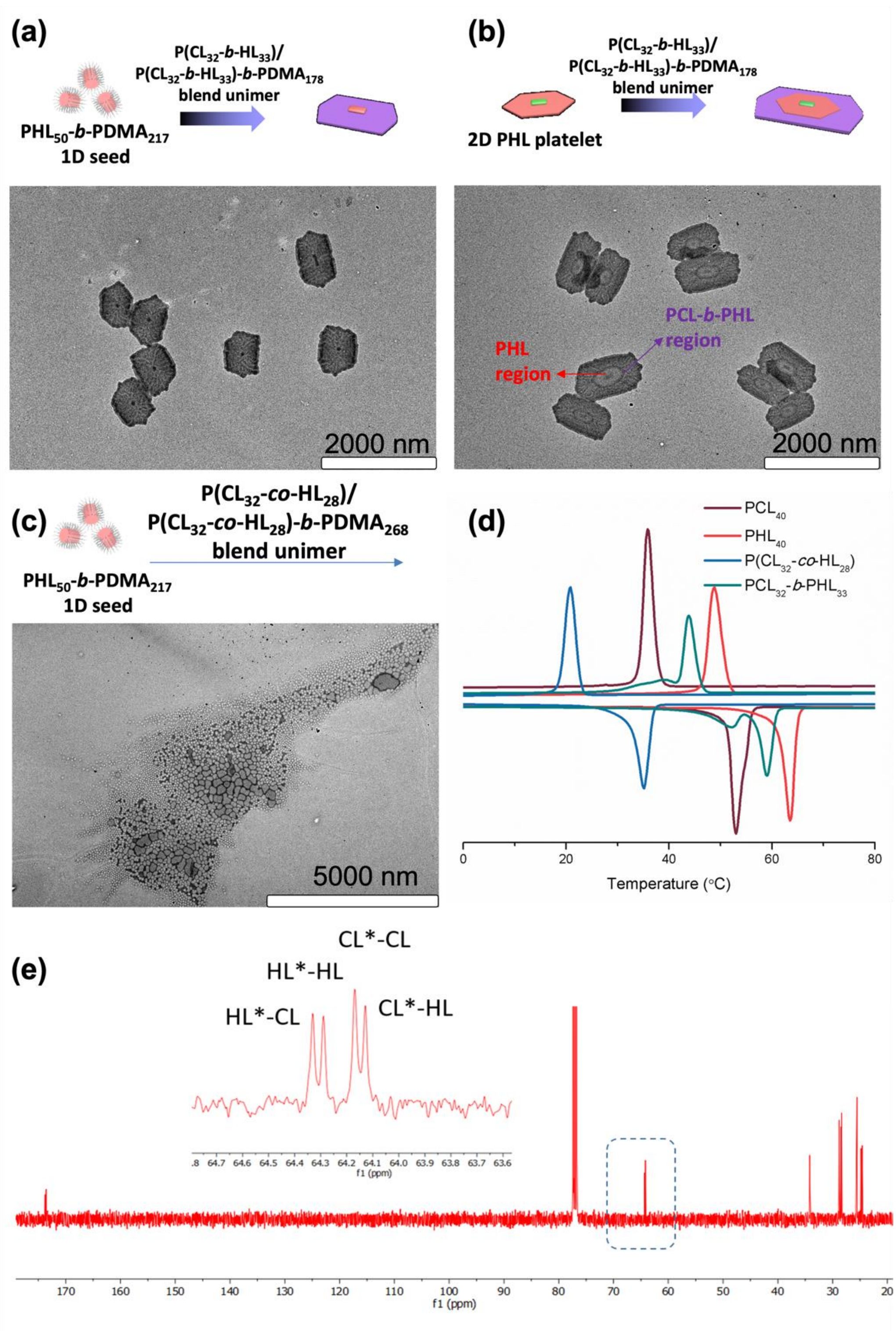

Supplementary Fig. 15. Effect of chain architectures of PCL/PHL copolymers on the growth from 1D PHL_{50}-*b*-$PDMA_{217}$ seed micelles or 2D PHL-based platelets. TEM micrographs of the structures formed upon adding $P(CL_{32}$-*b*-$HL_{33})/P(CL_{32}$-*b*-$HL_{33})$-*b*-$PDMA_{178}$ blend unimer to **(a)** 1D PHL_{50}-*b*-$PDMA_{217}$ seeds and **(b)** 2D PHL-based platelets; **(c)** TEM image of the structures formed upon adding $P(CL_{32}$-*co*-$HL_{28})/P(CL_{32}$-*co*-$HL_{28})$-*b*-$PDMA_{268}$ blend unimer into 1D PHL_{50}-*b*-$PDMA_{217}$ seeds; **(d)** DSC cooling and second heating scans of four different polymer cores; **(e)** ^{13}C NMR spectrum of $P(CL_{32}$-*co*-$HL_{28})$ in $CDCl_3$ shows the intensity of four peaks of HL*-CL, HL*-HL, CL*-CL and CL*-HL is similar, indicating a statistic random copolymer of $P(CL_{32}$-*co*-$HL_{28})$ core.

Due to a statistical random copolymer, the solubility of P(CL_{32}-*co*-HL_{28}) core in ethanol is significantly enhanced and meanwhile its crystallization ability is remarkably reduced, thus no epitaxial growth is observed (**Supplementary Fig. 15c**). Moreover, another P(CL_{32}-*co*-HL_{28})-*b*-$PDMA_{67}$ BCP with an evidently short corona length was prepared to obtain polydisperse cylinders by spontaneous nucleation in ethanol, but no cylinders were observed even after aging at room temperature for 2 months, indicating a retarded crystallization of P(CL_{32}-*co*-HL_{28}).

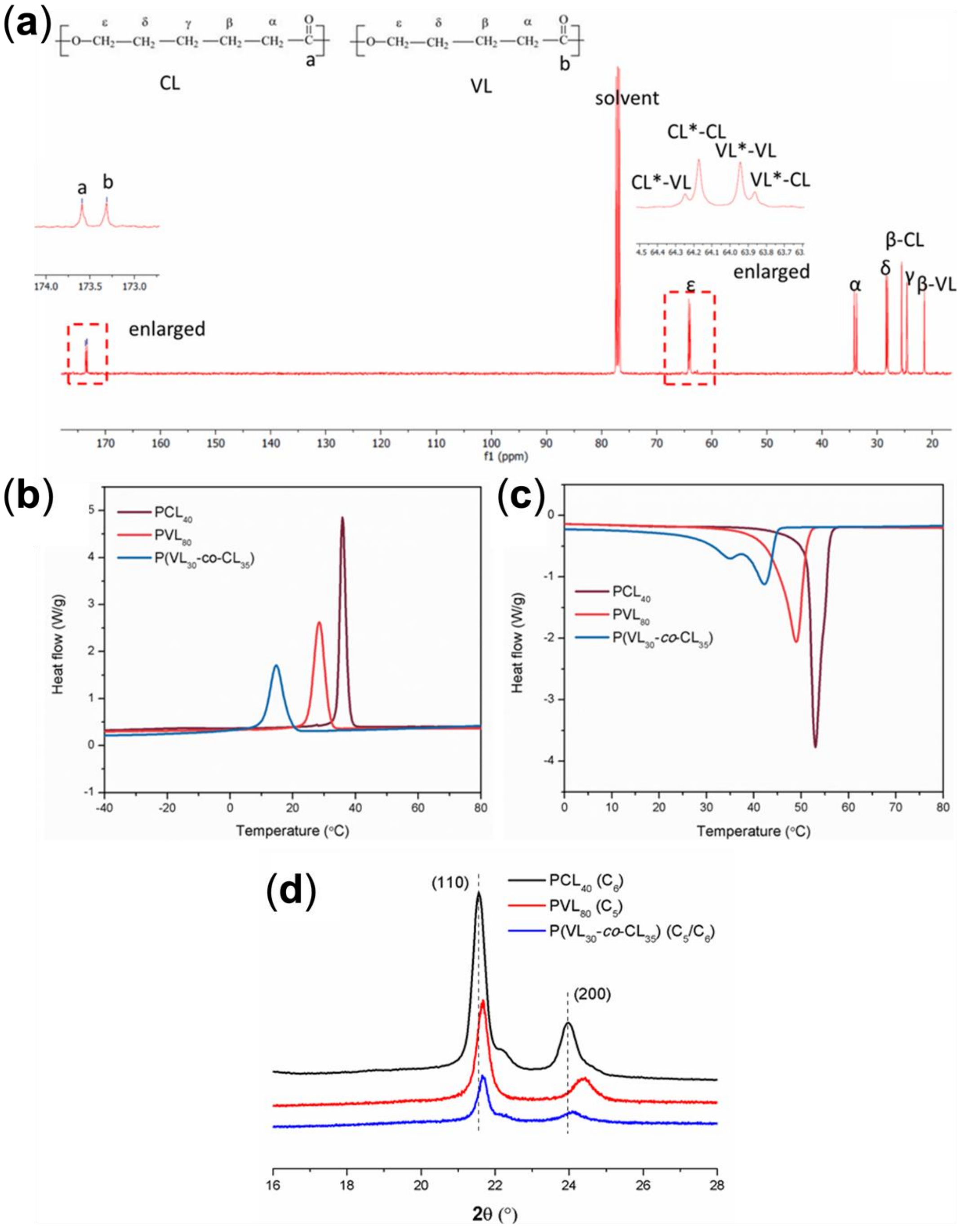

Supplementary Fig. 16. Chain architecture, thermal behaviour and crystalline forms of P(VL_{30}-*co*-CL_{35}) core. (a) ^{13}C NMR spectrum of P(VL_{30}-*co*-CL_{35}) core in $CDCl_3$ solvent; **(b)** DSC first cooling curves and **(c)** second heating curves of PVL_{80}, PCL_{40} and P(VL_{30}-*co*-CL_{35}); **(d)** WAXD curve of PVL_{80}, PCL_{40} and P(VL_{30}-*co*-CL_{35}).

The much stronger intensities of the CL*-CL and VL*-VL peaks in (A) compared with those of CL*-VL and VL*-CL indicate that the P(VL_{30}-*co*-CL_{35}) core presents a blocky chain architecture. DSC and WAXD results evidence the isomorphic crystallisation of the P(VL_{30}-*co*-CL_{35}) core, representing the cocrystallization of VL and CL units in the same unit cell since no separate PVL and PCL crystalline peaks are observed but instead only one peak for both (110) and (200) crystalline peaks was observed. The lower intensity of P(VL_{30}-*co*-CL_{35}) core from the WAXD curve also demonstrates a reduced crystallinity of the copolymer compared to the homopolymers.

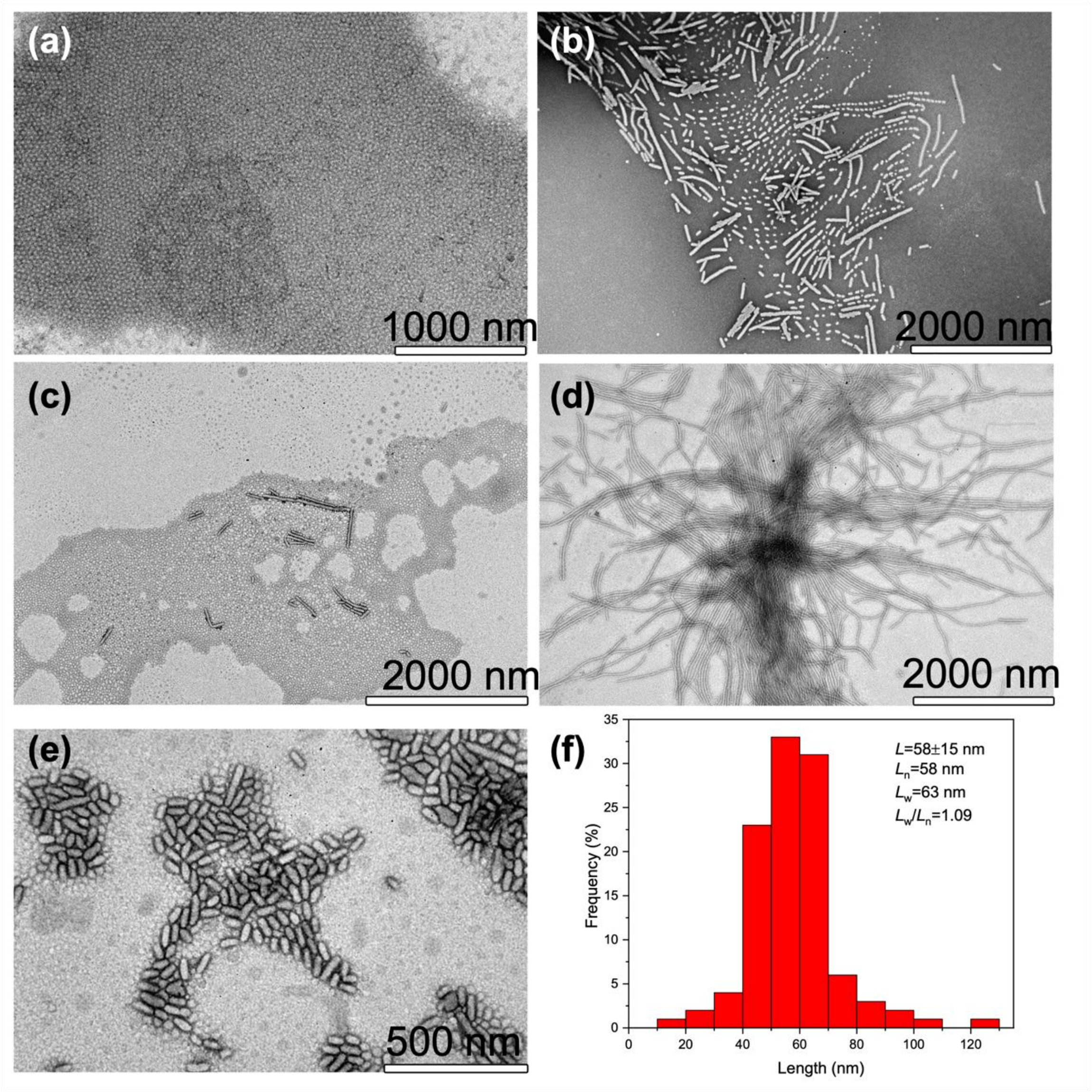

Supplementary Fig. 17. Effect of corona length on the self-assembly of BCPs with a P(VL_{30}-*co*-CL_{35}) core-forming component. Spontaneous nucleation and growth of P(VL_{30}-*co*-CL_{35})-*b*-$PDMA_{265}$ in ethanol at 5 mg/mL after heating at 70 °C for 3 h then subsequently cooling to room temperature (25 °C) for **(a)** 5 days and **(b)** 15 days. Spontaneous nucleation growth of P(VL_{30}-*co*-CL_{35})-*b*-$PDMA_{80}$ (short corona block) in ethanol at 5 mg/mL after heating at 70 °C for 3 h then subsequently cooling to room temperature (25 °C) for **(c)** 5 days and **(d)** 15 days. **(e)** Uniform 1D seeds of P(VL_{30}-*co*-CL_{35})-*b*-$PDMA_{80}$ prepared from the polydisperse cylinders in **(d)** after sonication at 0 °C for 20 min. **(f)** Corresponding length distribution of 1D seeds in **(e)** with average length of 58 nm.

Due to the reduced crystallization ability of the P(VL_{30}-*co*-CL_{35}) core, the corona polymerization degree of the corresponding BCP was reduced from 265 to 80 in order to prepare well-developed polydisperse cylinders in ethanol, thereby facilitating successful seed formation by sonication. We found that the P(VL_{30}-*co*-CL_{35})-*b*-$PDMA_{265}$ BCP only forms spherical micelles after aging for 5 days. Increasing the aging time for 15 days only yields some cylindrical micelles, indicating the crystallization rate of P(VL_{30}-*co*-CL_{35})-*b*-$PDMA_{265}$ is very slow. Decreasing the corona length can effectively increase the crystallization rate of P(VL_{30}-*co*-CL_{35})-*b*-$PDMA_{80}$ since well-developed polydisperse cylinders with several micrometers in length were obtained after aging for 15 days. In contrast, for spontaneous nucleation of PCL_{62}-*b*-$PDMA_{270}$ BCP with a long corona segment, well-developed polydisperse cylinders were formed after aging for 5 days (**Supplementary Fig. 2b**), suggesting that the crystallization rate of PCL is higher than that of P(VL_{30}-*co*-CL_{35}).

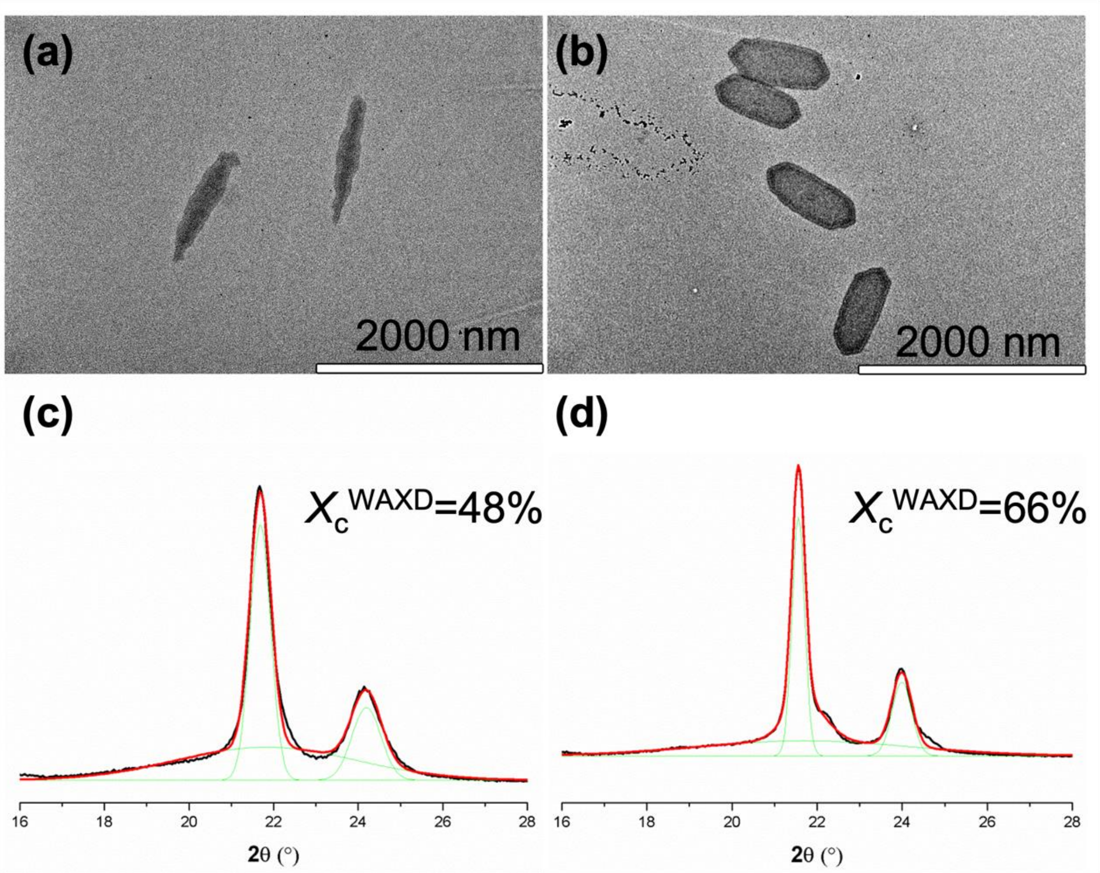

Supplementary Fig. 18. Evidence for reduced crystallinity of P(VL-*co*-CL) core-based 2D platelet micelles compared with that of PCL platelets. (a) Addition of P(VL_{30}-*co*-CL_{35})/P(VL_{30}-*co*-CL_{35})-*b*-$PDMA_{265}$ blend unimer (1:1, w/w) to 1D seeds of P(VL_{30}-*co*-CL_{35})-*b*-$PDMA_{80}$ with a mass ratio unimer-to-seed of 10; **(b)** addition of PCL_{40}/PCL_{62}-*b*-$PDMA_{270}$ blend unimer (1:1, w/w) to 1D seeds of P(VL_{30}-*co*-CL_{35})-*b*-$PDMA_{80}$ with a unimer-to-seed ratio of 10. Corresponding WAXD patterns of as-prepared 2D **(c)** P(VL-*co*-CL)-based and **(d)** PCL-based platelets with peak fitting included.

The crystallinity of both 2D platelets was calculated by separating the curve into three peaks including two crystalline peaks of (110) and (200), and one amorphous peak using following equation:

$X_c^{WAXD}=A_c/(A_a+A_c)$, where A_c is the total area of crystalline peaks and A_a is the area of amorphous peak, X_c^{WAXD} is the crystallinity obtained from WAXD result. The X_c^{WAXD} of 2D P(VL-*co*-CL) platelets is evidently lower than that of 2D PCL platelets, indicating a less ordered core of P(VL-*co*-CL).

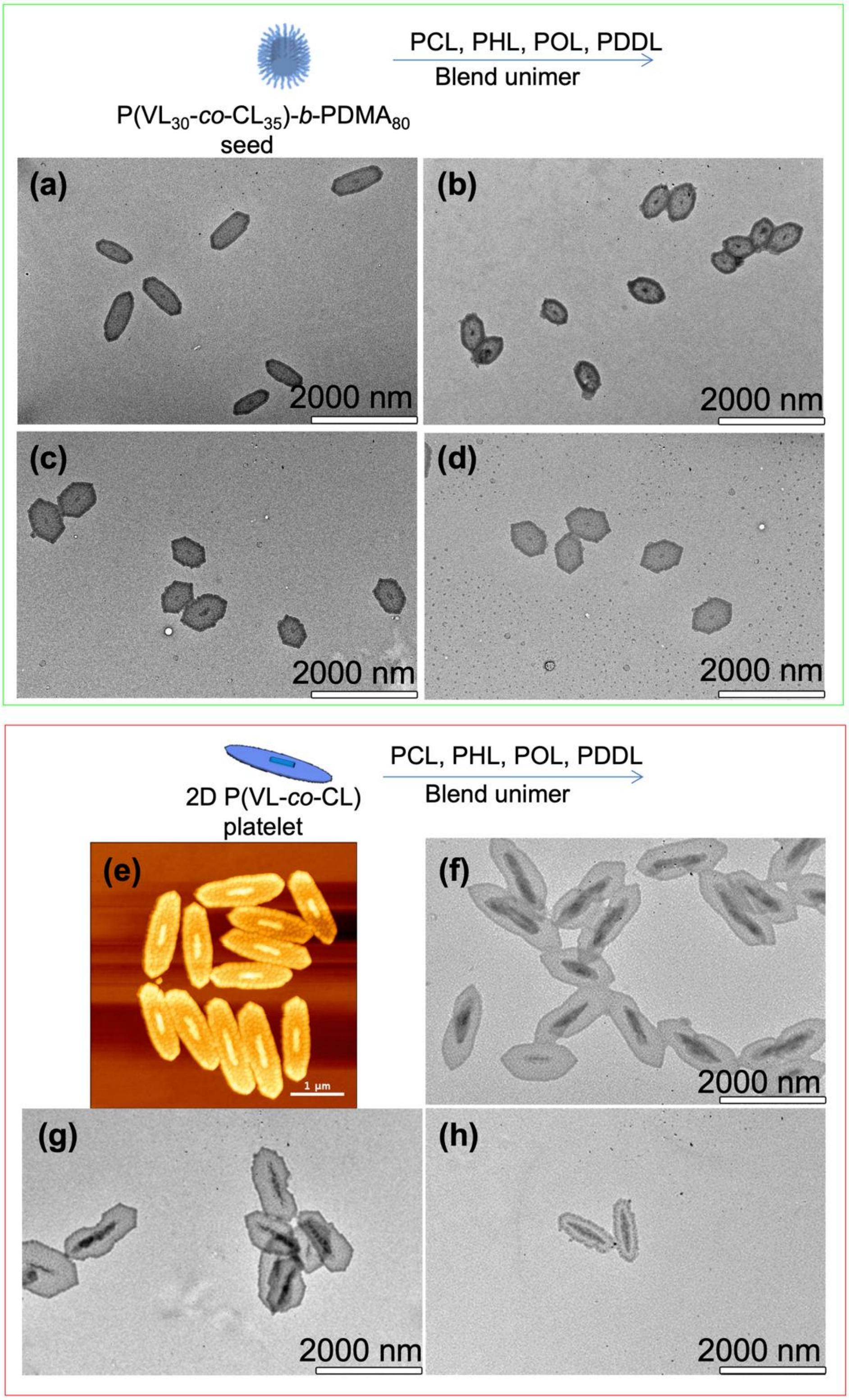

Supplementary Fig. 19. Sequential growth of the other core blend unimer from 1D P(VL$_{30}$-*co*-CL$_{35}$)-*b*-PDMA$_{80}$ seeds and 2D P(VL-*co*-CL)-based platelets. TEM morphologies for growth of **(a)** PCL$_{62}$/PCL$_{62}$-*b*-PDMA$_{270}$, **(b)** PHL$_{40}$/PHL$_{50}$-*b*-PDMA$_{217}$, **(c)** POL$_{55}$/POL$_{55}$-*b*-PDMA$_{280}$ and **(d)** PDDL$_{40}$/PDDL$_{40}$-*b*-PDMA$_{260}$ blend unimers (1:1, w/w, 0.1 mg, 10 mg/mL) from 1D seeds; **(e)** AFM height image of PCL$_{62}$/PCL$_{62}$-*b*-PDMA$_{270}$ and TEM images of **(f)** PHL$_{40}$/PHL$_{50}$-*b*-PDMA$_{217}$, **(g)** POL$_{55}$/POL$_{55}$-*b*-PDMA$_{280}$ and **(h)** PDDL$_{40}$/PDDL$_{40}$-*b*-PDMA$_{260}$ blend unimers (1:1, w/w, 0.06 mg, 10 mg/mL) grown from 2D P(VL-*co*-CL)-based platelets (0.02 mg, 1 mL).

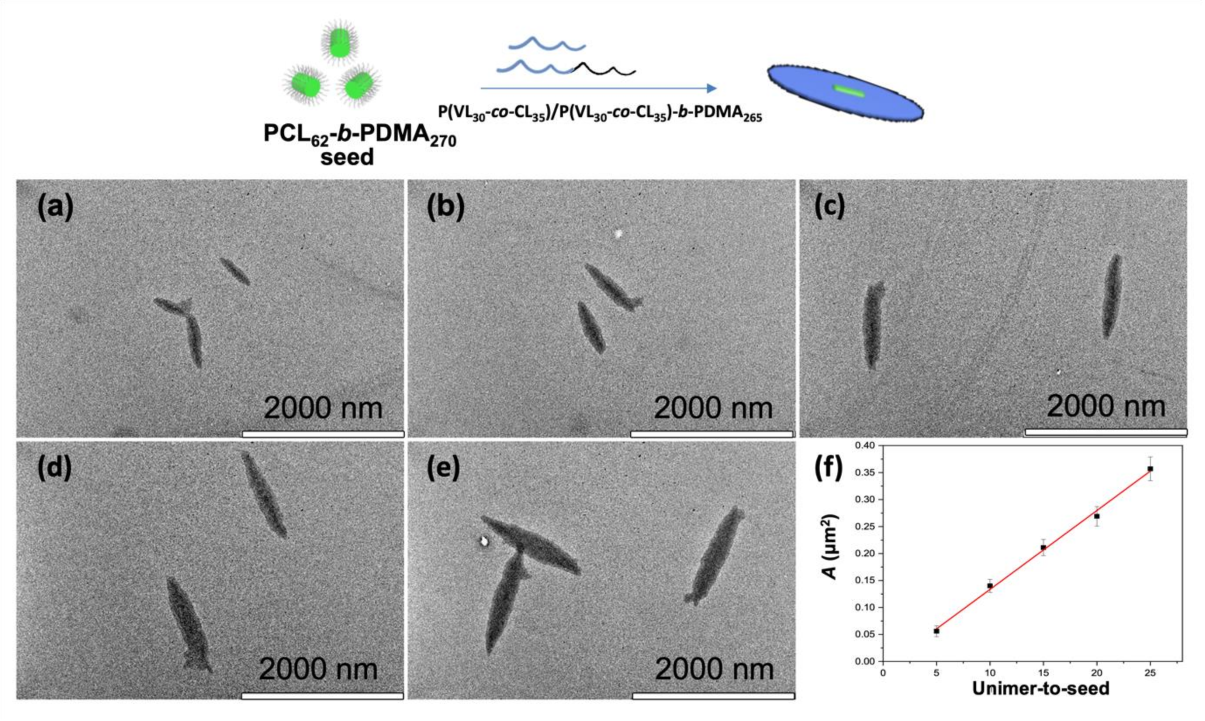

Supplementary Fig. 20. Living CDSA of P(VL$_{30}$-*co*-CL$_{35}$)/P(VL$_{30}$-*co*-CL$_{35}$)-*b*-PDMA$_{265}$ blend unimer (1:1, w/w) from a PCL$_{62}$-*b*-PDMA$_{270}$ seed. TEM micrographs of P(VL$_{30}$-*co*-CL$_{35}$)/P(VL$_{30}$-*co*-CL$_{35}$)-*b*-PDMA$_{265}$ blend unimer (1:1, w/w, 10 mg/mL in $CHCl_3$) grown from 1D seeds of PCL$_{62}$-*b*-PDMA$_{270}$ (0.01 mg, 1 mL) with mass ratio of unimer-to-seed of **(a)** 5, **(b)** 10, **(c)** 15, **(d)** 20 and **(e)** 25. **(f)** Plot of 2D platelet area against mass ratios of unimer-to-seed. Data are presented as mean values +/- standard deviation (n = 100).

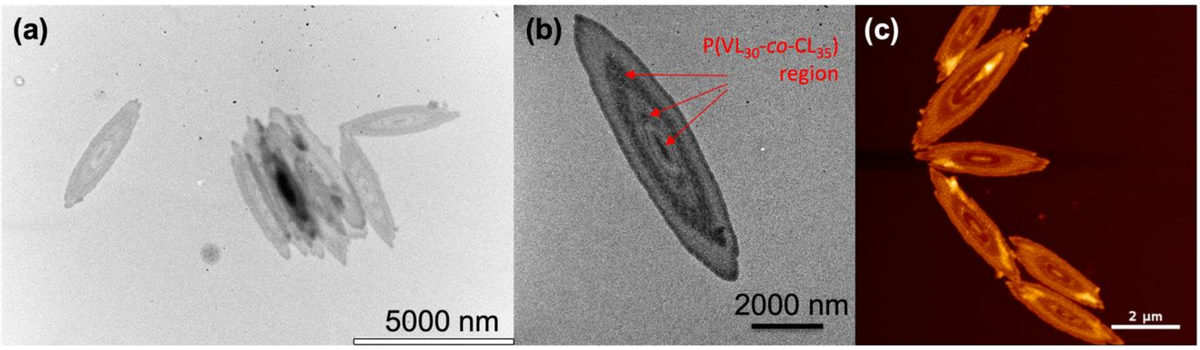

Supplementary Fig. 21. 2D Hexablock co-micelles. 2D block co-micelles prepared from sequential, alternate addition of P(VL_{30}-*co*-CL_{35})/P(VL_{30}-*co*-CL_{35})-*b*-$PDMA_{265}$ blend unimer (1:1, w/w) and PCL_{40}/PCL_{62}-*b*-$PDMA_{270}$ blend unimer (1:1, w/w) to the 1D seeds of PCL_{62}-*b*-$PDMA_{270}$. **(a)** Low magnification TEM image, **(b)** high magnification TEM image and **(c)** corresponding AFM height image of hexablock co-micelles.

It is observed that the P(VL-*co*-CL) regions exhibit significantly higher contrast in comparison to PCL region from the TEM observation, which is attributed to thicker layers of P(VL-*co*-CL) region. The AFM height image (**Fig. 3c, Fig. 4c**) indicates that the thickness of P(VL-*co*-CL) and PCL region is about 19.5 nm and 13.5 nm, respectively. However, removing the corona segment leads to the same thickness (~9 nm) of both regions (**Fig. 5b,c and Supplementary Fig. 23b,c**) and no significant contrast difference is observed in TEM image of the P(VL-*co*-CL) and PCL regions. These observations elucidate that the intensity contrast is not related to the core crystallinity but more relevant to the thickness of the platelets. Therefore, we believe the darker phase of P(VL-*co*-CL) region is attributed to a thicker layer of PDMA domain tethered on both surfaces of P(VL-*co*-CL) region, which is caused by a different chain packing between PCL and P(VL-*co*-CL) as revealed by WAXD result (**Supplementary Fig. 16d and Supplementary Fig. 18c,d**).

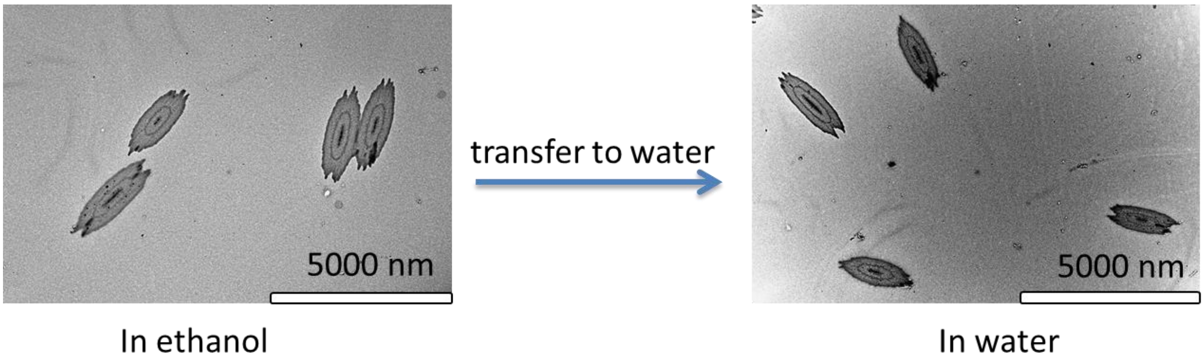

Supplementary Fig. 22. Stability of the segmented ABC triblock co-micelles (A = P(VL-*co*-CL) (inner core region), B = PCL (central core region), C = PHL (outer core region)) in different solvents. TEM morphologies of the triblock co-micelles after transferring from ethanol to water. No morphological changes are observed after solvent change, which implies the block co-micelles are also stable in water.

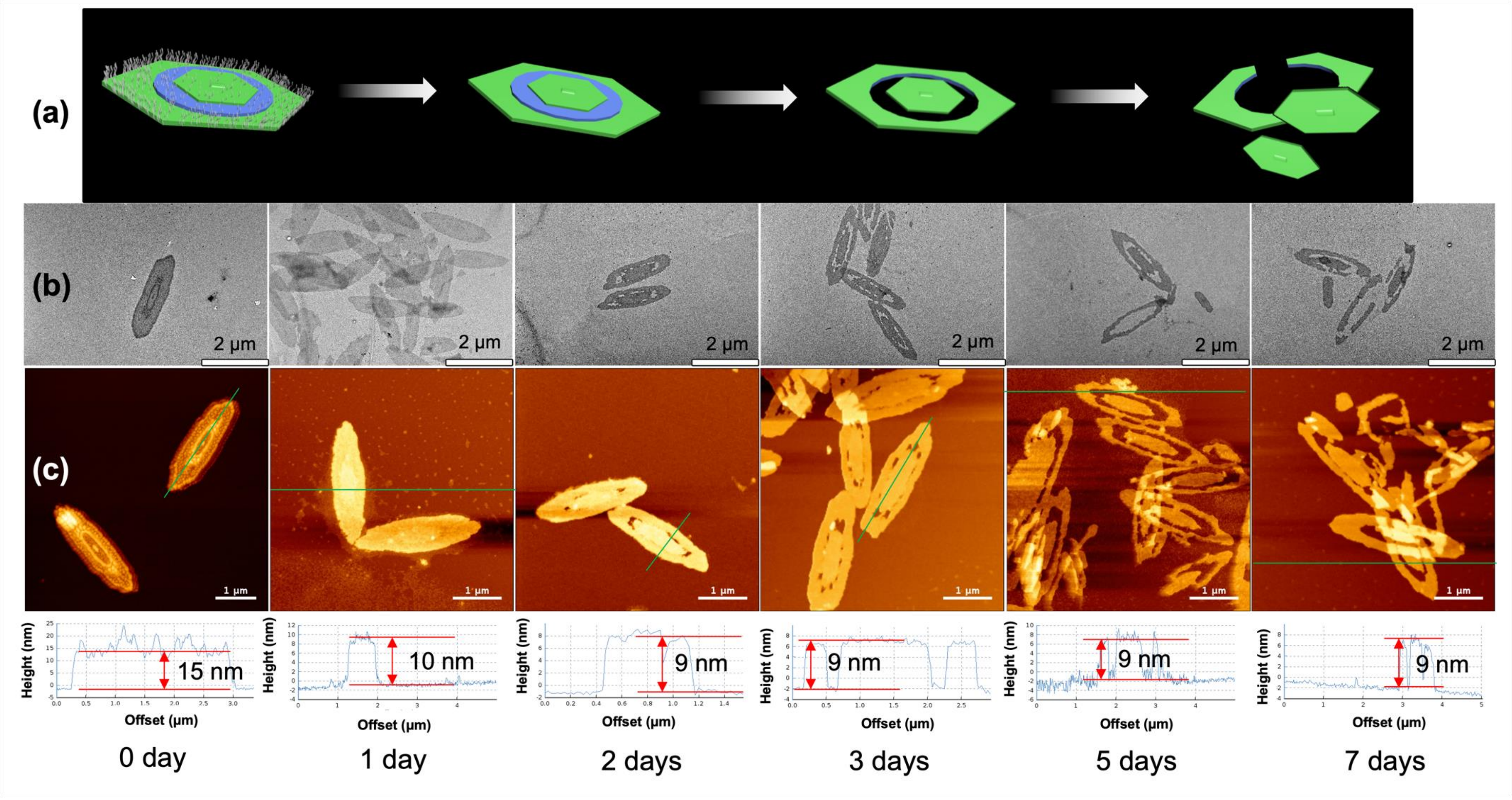

Supplementary Fig. 23. Selective degradation of 2D triblock co-micelles of PCL-P(VL-*co*-CL)-PCL segmented ABA triblock co-micelles (A = PCL (inner and outer core region), B = P(VL-*co*-CL) (central core region)). Time-resolved degradation for the triblock co-micelles of PCL-P(VL-*co*-CL)-PCL in the presence of 1 M KOH aqueous solution. **(a)** Schematic illustration of the degradation process; **(b)** the TEM morphologies and **(c)** corresponding AFM height images and the height profiles of the line indicated in the **(c)** of the ABA triblock co-micelles at different degradation time.

(a)

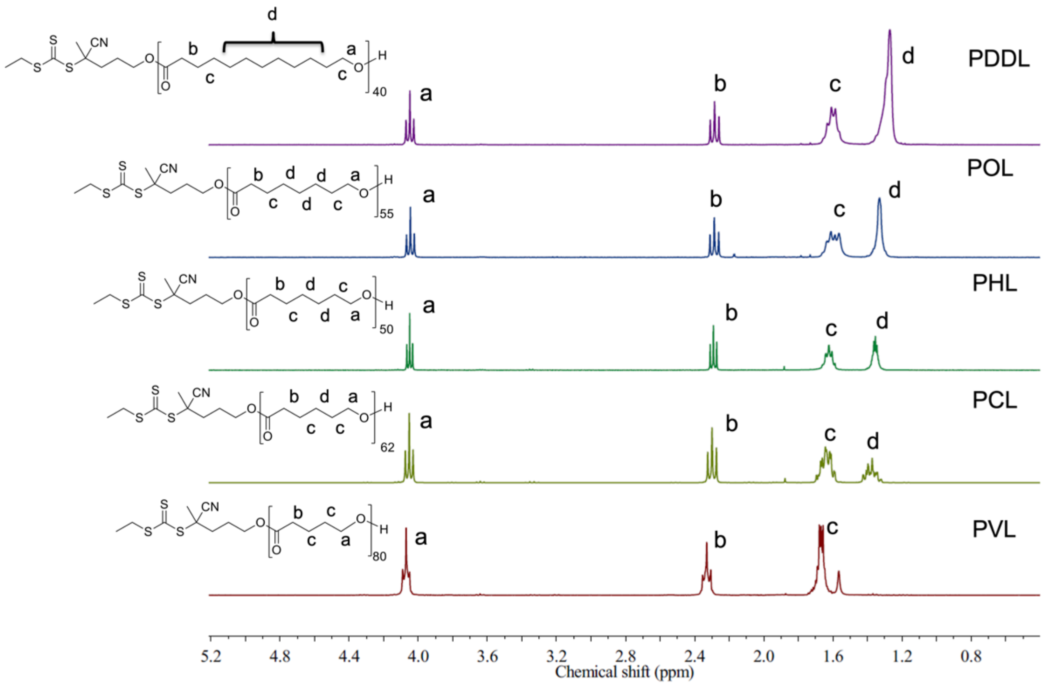

(b)

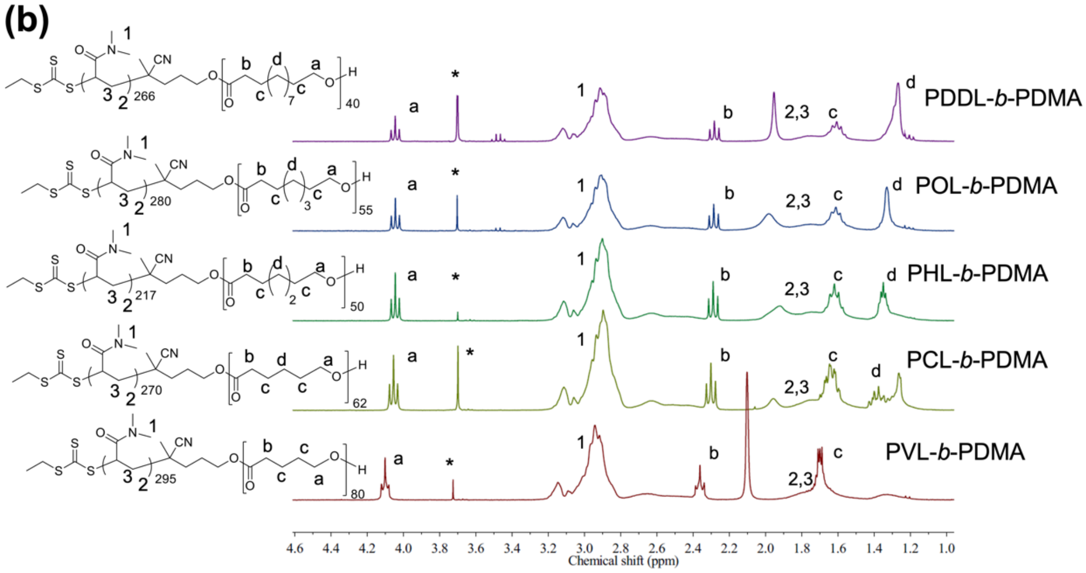

Supplementary Fig. 24. Molecular characterization of different polymers. ^{1}H NMR spectra of **(a)** different cores and **(b)** corresponding BCPs in $CDCl_3$. * represents the residual solvent.

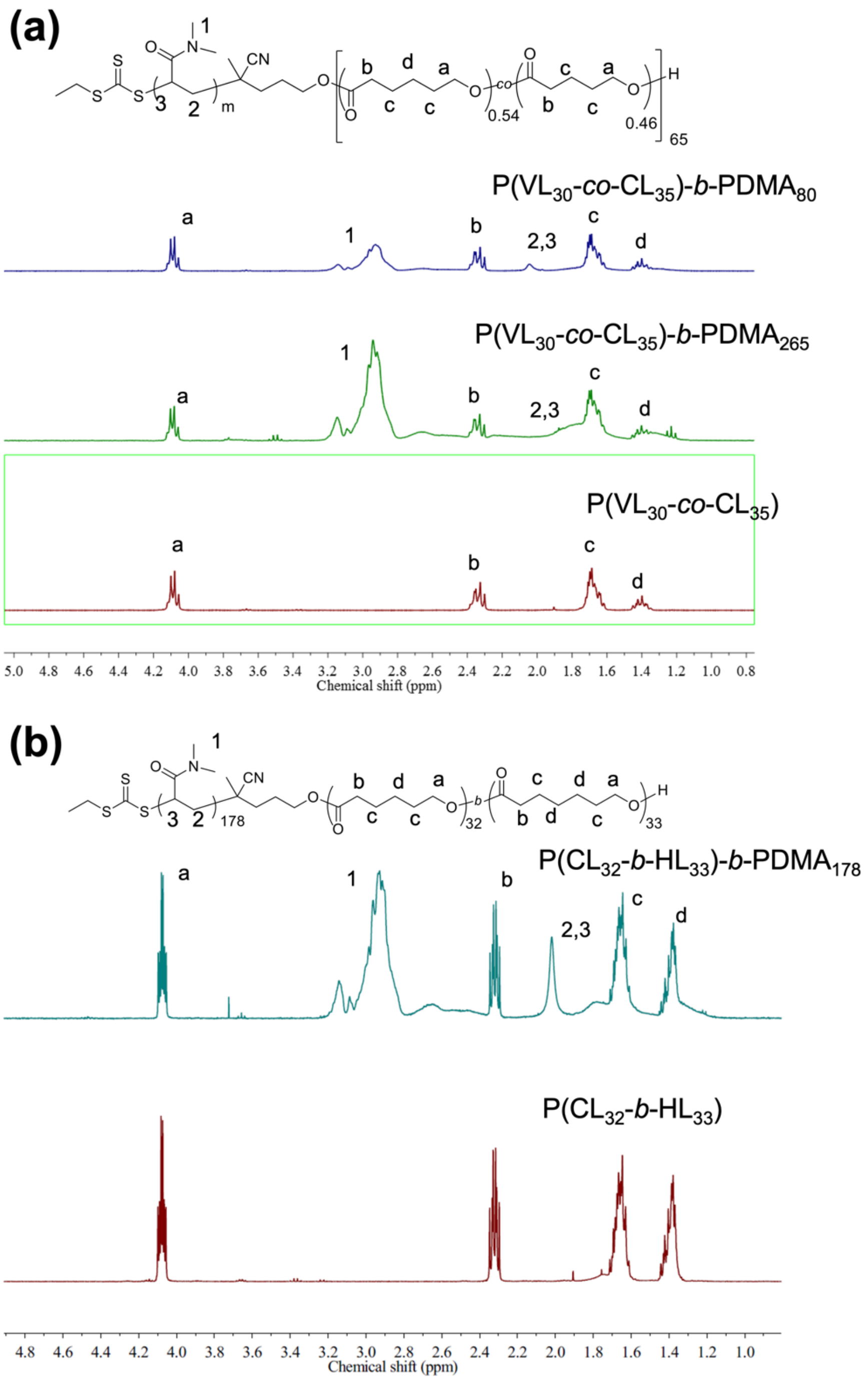

Supplementary Fig. 25. Molecular characterization of different polymers. ^{1}H NMR spectra of **(a)** P(VL-*co*-CL) based core and corresponding BCPs and **(b)** P(CL-*b*-HL) based cores and corresponding BCPs in $CDCl_3$.

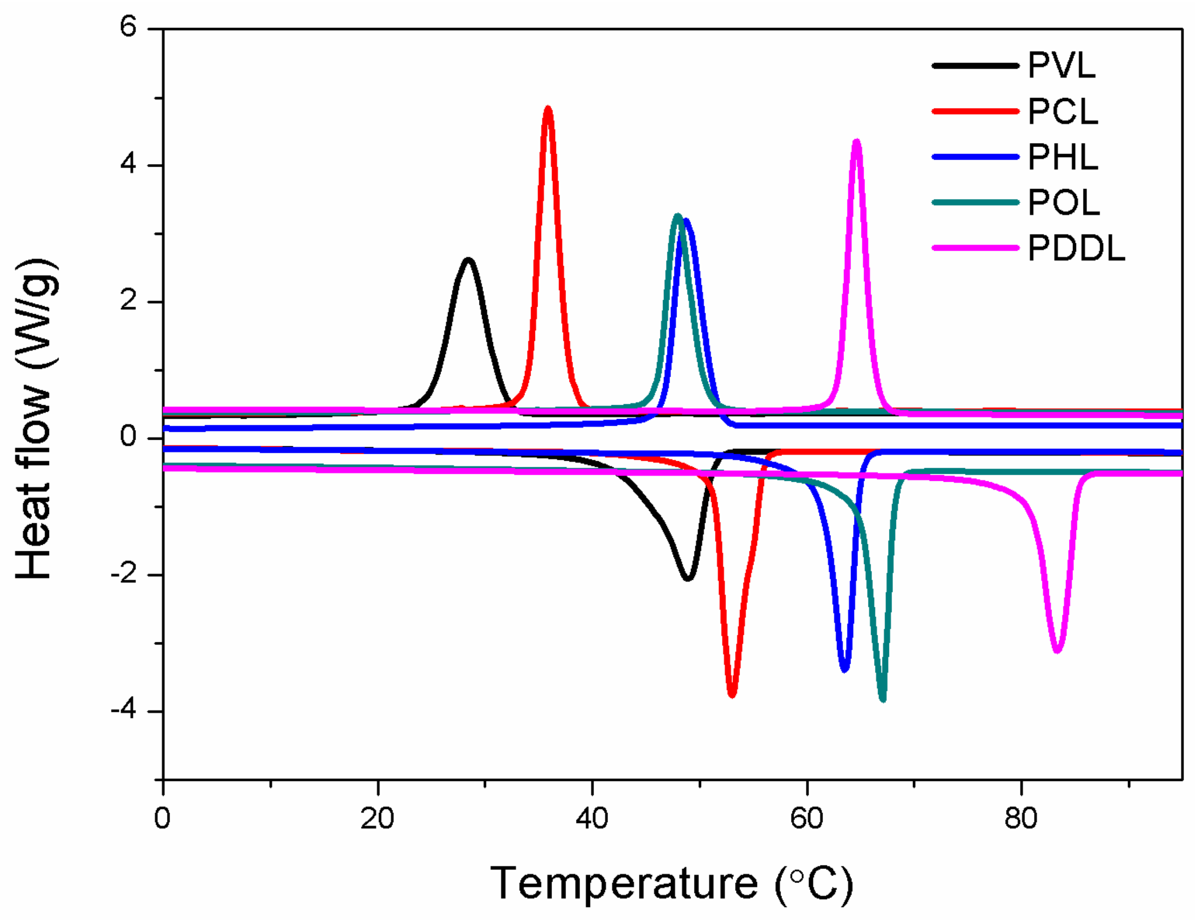

Supplementary Fig. 26. Thermal behaviour of different homopolymers. DSC second cooling and heating scans of different cores. Both the heating and cooling rates are 10 °C/min.

References

1. X. Zhuang, Y. Mai, D. Wu, F. Zhang, X. Feng, Two-dimensional soft nanomaterials: A fascinating world of materials. Adv. Mater. 27, 403-427 (2015). doi: 10.1002/adma.201401857.

2. C. Zhou, Y. Zhang, Y. Dong, F. Wu, D. Wang, L. Xin, D. Liu, Precisely controlled 2D free-floating nanosheets of amphiphilic molecules through frame-guided assembly. Adv. Mater. 28, 9819-9823 (2016). doi: 10.1002/adma.201603210.

3. S. Agbolaghi, S. Abbaspoor, F. Abbasi, A comprehensive review on polymer single crystals-from fundamental concepts to applications. Prog. Polym. Sci. 81, 22-79 (2018). doi: 10.1016/j.progpolymsci.2017.11.006.

4. S. Yang, S.-Y. Kang, T.-L. Choi, Morphologically tunable square and rectangular nanosheets of a simple conjugated homopolymer by changing solvents. J. Am. Chem. Soc. 141, 19138-19143 (2019). doi: 10.1021/jacs.9b10904.

5. L. Kang, A. Chao, M. Zhang, T. Yu, J. Wang, Q. Wang, H. Yu, N. Jiang, D. Zhang, Modulating the molecular geometry and solution self-assembly of amphiphilic polypeptoid block copolymers by side chain branching pattern. J. Am. Chem. Soc. 143, 5890-5902 (2021). doi: 10.1021/jacs.1c01088.

6. A. K. Pearce, T. R. Wilks, M. C. Arno, R. K. O'Reilly, Synthesis and applications of anisotropic nanoparticles with precisely defined dimensions. Nat. Rev. Chem. 5, 21-45 (2021). doi: 10.1038/s41570-020-00232-7.

7. Z. M. Hudson, C. E. Boott, M. E. Robinson, P. A. Rupar, M. A. Winnik, I. Manners, Tailored hierarchical micelle architectures using living crystallization-driven self-assembly in two dimensions. Nat. Chem. 6, 893-898 (2014). doi: 10.1038/nchem.2038.

8. M. Inam, J. R. Jones, M. M. Perez-Madrigal, M. C. Arno, A. P. Dove, R. K. O'Reilly, Controlling the size of two-dimensional polymer platelets for water-in-water emulsifiers. ACS Cental Sci. 4, 63-70 (2018). doi: 10.1021/acscentsci.7b00436.

9. W. Y. Chen, C. Y. Li, J. X. Zheng, P. Huang, L. Zhu, Q. Ge, R. P. Quirk, B. Lotz, L. Deng, C. Wu, E. L. Thomas, S. Z. D. Cheng, "Chemically shielded" poly(ethylene oxide) single crystal growth and construction of channel-wire arrays with chemical and geometric recognitions on a submicrometer scale. Macromolecules 37, 5292-5299 (2004). doi: 10.1021/ma0493325.

10. X. He, M.-S. Hsiao, C. E. Boott, R. L. Harniman, A. Nazemi, X. Li, M. A. Winnik, I. Manners, Two-dimensional assemblies from crystallizable homopolymers with charged termini. Nat. Mater. 16, 481-489 (2017). doi: 10.1038/nmat4837.

11. B. Fan, R. Y. Wang, X. Y. Wang, J. T. Xu, B. Y. Du, Z. Q. Fan, Crystallization-driven co-assembly of micrometric polymer hybrid single crystals and nanometric crystalline micelles. Macromolecules 50, 2006-2015 (2017). doi: 10.1021/acs.macromol.7b00105.

12. F. Xu, P. Zhang, J. Zhang, C. Yu, D. Yan, Y. Mai, Crystallization-driven two-dimensional self-assembly of amphiphilic PCL-b-PEO coated gold nanoparticles in aqueous solution. ACS Macro Lett. 7, 1062-1067 (2018). doi: 10.1021/acsmacrolett.8b00383.

13. L. Han, M. Wang, X. Jia, W. Chen, H. Qian, F. He, Uniform two-dimensional square assemblies from conjugated block copolymers driven by π-π interactions with controllable sizes. Nat. Commun. 9, 865 (2018). doi: 10.1038/s41467-018-03195-y.

14. A. D. Merg, E. van Genderen, A. Bazrafshan, H. Su, X. Zuo, G. Touponse, T. B. Blum, K. Salaita, J. P. Abrahams, V. P. Conticello, Seeded heteroepitaxial growth of crystallizable collagen triple helices: Engineering multifunctional two-dimensional core-shell nanostructures. J. Am. Chem. Soc. 141, 20107-20117 (2019). doi: 10.1021/jacs.9b09335.

15. R. Qi, Y. Zhu, L. Han, M. Wang, F. He, Rectangular platelet micelles with controlled aspect ratio by hierarchical self-assembly of poly(3-hexylthiophene)-b-poly(ethylene glycol). Macromolecules 53, 6555-6565 (2020). doi: 10.1021/acs.macromol.0c01092.

16. J. Sun, Z. Wang, C. Zhu, M. Wang, Z. Shi, Y. Wei, X. Fu, X. Chen, R. N. Zuckermann, Hierarchical supramolecular assembly of a single peptoid polymer into a planar nanobrush with two distinct molecular packing motifs. Proc. Natl. Acad. Sci. USA 117, 31639-31647 (2020). doi: 10.1073/pnas.2011816117.

17. S. Yang, S.-Y. Kang, T.-L. Choi, Semi-conducting 2D rectangles with tunable length via uniaxial living crystallization-driven self-assembly of homopolymer. Nat. Commun. 12, 2602-2602 (2021). doi: 10.1038/s41467-021-22879-6.

18. S. Ganda, C. K. Wong, M. H. Stenzel, Corona-loading strategies for crystalline particles made by living crystallization-driven self-assembly. Macromolecules 54, 6662-6669 (2021). doi: 10.1021/acs.macromol.1c00643.

19. S. Ganda, M. H. Stenzel, Concepts, fabrication methods and applications of living crystallization-driven self-assembly of block copolymers. Prog. Polym. Sci. 101, 101195 (2020). doi: 10.1016/j.progpolymsci.2019.101195.

20. H. Qiu, Y. Gao, C. E. Boott, O. E. C. Gould, R. L. Harniman, M. J. Miles, S. E. D. Webb, M. A. Winnik, I. Manners, Uniform patchy and hollow rectangular platelet micelles from crystallizable polymer blends. Science 352, 697-701 (2016). doi: 10.1126/science.aad9521.

21. D. Tao, C. Feng, Y. Cui, X. Yang, I. Manners, M. A. Winnik, X. Huang, Monodisperse fiber-like micelles of controlled length and composition with an oligo(p-phenylenevinylene) core via "living" crystallization-driven self-assembly. J. Am. Chem. Soc. 139, 7136-7139 (2017). doi: 10.1021/jacs.7b02208.

22. J. Nie, Z. Wang, X. Huang, G. Lu, C. Feng, Uniform continuous and segmented nanofibers containing a π-conjugated oligo(p-phenylene ethynylene) core via "living" crystallization-driven self-assembly: Importance of oligo(p-phenylene ethynylene) chain length. Macromolecules 53, 6299-6313 (2020). doi: 10.1021/acs.macromol.0c01199.

23. C. Hils, J. Schmelz, M. Drechsler, H. Schmalz, Janus micelles by crystallization-driven self-assembly of an amphiphilic, double-crystalline triblock terpolymer. J. Am. Chem. Soc. 143, 15582-15586 (2021). doi: 10.1021/jacs.1c08076.

24. J. R. Finnegan, E. H. Pilkington, K. Alt, M. A. Rahim, S. J. Kent, T. P. Davis, K. Kempe, Stealth nanorods via the aqueous living crystallisation-driven self-assembly of poly(2-oxazoline)s. Chem. Sci. 12, 7350-7360 (2021). doi: 10.1039/d1sc00938a.

25. W. Zhang, W. Jin, T. Fukushima, A. Saeki, S. Seki, T. Aida, Supramolecular linear heterojunction composed of graphite-like semiconducting nanotubular segments. Science 334, 340-343 (2011). doi: 10.1126/science.1210369.

26. J. Nie, D. Tao, X. Huang, G. Lu, C. Feng, Uniform nanowires containing a heterogeneous π-conjugated core of controlled length, composition and morphology. Chem. Eur. J. 27, 8479-8483 (2021). doi: 10.1002/chem.202100940.

27. T. Gadt, N. S. Ieong, G. Cambridge, M. A. Winnik, I. Manners, Complex and hierarchical micelle architectures from diblock copolymers using living, crystallization-driven polymerizations. Nat. Mater. 8, 144-150 (2009). doi: 10.1038/NMAT2356.

28. A. Nazemi, X. He, L. R. MacFarlane, R. L. Harniman, M.-S. Hsiao, M. A. Winnik, C. F. J. Faul, I. Manners, Uniform "patchy" platelets by seeded heteroepitaxial growth of crystallizable polymer blends in two dimensions. J. Am. Chem. Soc. 139, 4409-4417 (2017). doi: 10.1021/jacs.6b12503.

29. Y. Zhang, S. Pearce, J.-C. Eloi, R. L. Harniman, J. Tian, C. Cordoba, Y. Kang, T. Fukui, H. Qiu, A. Blackburn, R. M. Richardson, I. Manners, Dendritic micelles with controlled branching and sensor applications. J. Am. Chem. Soc. 143, 5805-5814 (2021). doi: 10.1021/jacs.1c00770.

30. L. Sun, A. Pitto-Barry, N. Kirby, T. L. Schiller, A. M. Sanchez, M. A. Dyson, J. Sloan, N. R. Wilson, R. K. O'Reilly, A. P. Dove, Structural reorganization of cylindrical nanoparticles triggered by polylactide stereocomplexation. Nat. Commun. 5, 5747 (2014). doi: 10.1038/ncomms6746.

31. M. C. Arno, M. Inam, Z. Coe, G. Cambridge, L. J. Macdougall, R. Keogh, A. P. Dove, R. K. O'Reilly, Precision epitaxy for aqueous 1D and 2D poly(ε-caprolactone) assemblies. J. Am. Chem. Soc. 139, 16980-16985 (2017). doi: 10.1021/jacs.7b10199.

32. Y. He, J.-C. Eloi, R. L. Harniman, R. M. Richardson, G. R. Whittell, R. T. Mathers, A. P. Dove, R. K. O'Reilly, I. Manners, Uniform biodegradable fiber-like micelles and block comicelles via "living" crystallization-driven self-assembly of poly(L-lactide) block copolymers: The importance of reducing unimer self-nucleation via hydrogen bond disruption. J. Am. Chem. Soc. 141, 19088-19098 (2019). doi: 10.1021/jacs.9b09885.

33. Z. Tong, Y. Su, Y. Jiang, Y. Xie, S. Chen, R. K. O'Reilly, Spatially restricted templated growth of poly(ε-caprolactone) from carbon nanotubes by crystallization-driven self-assembly. Macromolecules 54, 2844-2851 (2021). doi: 10.1021/acs.macromol.0c02739.

34. M. Bartnikowski, T. R. Dargaville, S. Ivanovski, D. W. Hutmacher, Degradation mechanisms of polycaprolactone in the context of chemistry, geometry and environment. Prog. Polym. Sci. 96, 1-20 (2019). doi: 10.1016/j.progpolymsci.2019.05.004.

35. J. C. Wittmann, A. M. Hodge, B. Lotz, Epitaxial crystallization of polymers onto benzoic acid: Polyethylene and paraffins, aliphatic polyesters, and polyamides. J. Polym. Sci., Polym. Phys. Ed. 21, 2495-2509 (1983). doi: https://doi.org/10.1002/pol.1983.180211207.

36. P. Damman, S. Coppée, V. M. Geskin, R. Lazzaroni, What is the mechanism of oriented crystal growth on rubbed polymer substrates? Topography vs epitaxy. J. Am. Chem. Soc. 124, 15166-15167 (2002). doi: 10.1021/ja027145l.

37. L. Li, J. Hu, Y. Li, Q. Huang, X. Sun, S. Yan, Evidence for the soft and hard epitaxies of poly(L-lactic acid) on an oriented polyethylene substrate and their dependence on the crystallization temperature. Macromolecules 53, 1745-1751 (2020). doi: 10.1021/acs.macromol.9b02598.

38. L. Li, C. Y. Li, C. Ni, Polymer crystallization-driven, periodic patterning on carbon nanotubes. J. Am. Chem. Soc. 128, 1692-1699 (2006). doi: 10.1021/ja056923h.

39. R. P. Brannigan, A. P. Dove, Synthesis, properties and biomedical applications of hydrolytically degradable materials based on aliphatic polyesters and polycarbonates. Biomater. Sci. 5, 9-21 (2017). doi: 10.1039/C6BM00584E.

40. A. J. Greso, P. J. Phillips, The role of secondary nucleation in epitaxial growth: The template model. Polymer 35, 3373-3376 (1994). doi: https://doi.org/10.1016/0032-3861(94)90897-4.

41. Y. Zheng, P. Pan, Crystallization of biodegradable and biobased polyesters: Polymorphism, cocrystallization, and structure-property relationship. Prog. Polym. Sci. 109, 101291 (2020). doi: 10.1016/j.progpolymsci.2020.101291.

42. X. He, Y. He, M.-S. Hsiao, R. L. Harniman, S. Pearce, M. A. Winnik, I. Manners, Complex and hierarchical 2D assemblies via crystallization-driven self-assembly of poly(L-lactide) homopolymers with charged termini. J. Am. Chem. Soc. 139, 9221-9228 (2017). doi: 10.1021/jacs.7b03172.

43. J. A. Wilson, S. A. Hopkins, P. M. Wright, A. P. Dove, 'Immortal' ring-opening polymerization of omega-pentadecalactone by $Mg(BHT)_2(THF)_2$. *Polym. Chem.* 5, 2691-2694 (2014). doi: 10.1039/c4py00034j.

44. L. van der Mee, F. Helmich, R. de Bruijn, J. A. J. M. Vekemans, A. R. A. Palmans, E. W. Meijer, Investigation of lipase-catalyzed ring-opening polymerizations of lactones with various ring sizes: Kinetic evaluation. *Macromolecules* **39**, 5021-5027 (2006). doi: 10.1021/ma060668j.

45. J. A. Wilson, S. A. Hopkins, P. M. Wright, A. P. Dove, Synthesis of ω-pentadecalactone copolymers with independently tunable thermal and degradation behavior. *Macromolecules* 48, 950-958 (2015). doi: 10.1021/ma5022049.
